# Cytometry profiling of *ex vivo* recall responses to *Coxiella burnetii* in previously naturally exposed individuals reveals long-term changes in both adaptive and innate immune cellular compartments

**DOI:** 10.3389/fimmu.2023.1249581

**Published:** 2023-10-11

**Authors:** Susan Raju Paul, Anja Scholzen, Patrick M. Reeves, Robert Shepard, Joshua M. Hess, Richard K. Dzeng, Skylar Korek, Anja Garritsen, Mark C. Poznansky, Ann E. Sluder

**Affiliations:** ^1^ Vaccine and Immunotherapy Center, Massachusetts General Hospital, Boston, MA, United States; ^2^ InnatOss Laboratories B.V., Oss, Netherlands

**Keywords:** *Coxiella burnetii*, Q fever, mass cytometry, cytokines, immune profiling, human, innate, cellular immunity

## Abstract

**Introduction:**

Q fever, caused by the intracellular bacterium *Coxiella burnetii*, is considered an occupational and biodefense hazard and can result in debilitating long-term complications. While natural infection and vaccination induce humoral and cellular immune responses, the exact nature of cellular immune responses to *C. burnetii* is incompletely understood. The current study seeks to investigate more deeply the nature of long-term cellular recall responses in naturally exposed individuals by both cytokine release assessment and cytometry profiling.

**Methods:**

Individuals exposed during the 2007-2010 Dutch Q fever outbreak were grouped in 2015, based on a *C. burnetii*-specific IFNγ release assay (IGRA), serological status, and self-reported clinical symptoms during initial infection, into asymptomatic IGRA-negative/seronegative controls, and three IGRA-positive groups (seronegative/asymptomatic; seropositive/asymptomatic and seropositive/symptomatic). Recall responses following *in vitro* re-stimulation with heat-inactivated *C. burnetii* in whole blood, were assessed in 2016/2017 by cytokine release assays (n=55) and flow cytometry (n=36), and in blood mononuclear cells by mass cytometry (n=36).

**Results:**

Cytokine release analysis showed significantly elevated IL-2 responses in all seropositive individuals and elevated IL-1β responses in those recovered from symptomatic infection. Comparative flow cytometry analysis revealed significantly increased IFNγ, TNFα and IL-2 recall responses by CD4 T cells and higher IL-6 production by monocytes from symptomatic, IGRA-positive/seropositive individuals compared to controls. Mass cytometry profiling and unsupervised clustering analysis confirmed recall responses in seropositive individuals by two activated CD4 T cell subsets, one characterized by a strong Th1 cytokine profile (IFNγ^+^IL-2^+^TNFα^+^), and identified *C. burnetii*-specific activation of CD8 T cells in all IGRA-positive groups. Remarkably, increased *C. burnetii*-specific responses in IGRA-positive individuals were also observed in three innate cell subpopulations: one characterized by an IFNγ^+^IL-2^+^TNFα^+^ Th1 cytokine profile and lack of canonical marker expression, and two IL-1β-, IL-6- and IL-8-producing CD14^+^ monocyte subsets that could be the drivers of elevated secretion of innate cytokines in pre-exposed individuals.

**Discussion:**

These data highlight that there are long-term increased responses to *C. burnetii* in both adaptive and innate cellular compartments, the latter being indicative of trained immunity. These findings warrant future studies into the protective role of these innate responses and may inform future Q fever vaccine design.

## Introduction

Q fever is a zoonotic disease caused by the obligate intracellular gram-negative coccobacillus, *Coxiella burnetii*, and transmitted to humans through aerosols from infected ruminants such as goats, sheep, and cattle (1). *C. burnetii* exhibits significant resilience even in harsh environments (2) and is highly infectious with an ID50 of one bacterium (3). Consequently, *C. burnetii* is classified as a category B bioterrorism agent (4). Acute Q fever is asymptomatic in most infected individuals and, when symptomatic, presents largely with flu-like symptoms (1). Therefore, infection with *C. burnetii* is likely significantly underreported as observed in the largest reported outbreak of Q fever, which occurred in the Netherlands from 2007-2010. Although only 3500 infections were officially reported (5, 6), a survey of serological data suggested that there were an estimated 40,000 infections in the region at the center of the epidemic alone (7). While acute infection is often self-limiting and readily treatable with antibiotics such as tetracyclines, long-term complications of infection are common; 10-20% of patients with acute Q fever later develop Q fever fatigue syndrome, and 1-5% of infected individuals (often with other comorbidities) progress to chronic Q fever, which manifests as endocarditis, aneurysms, or vascular infections (1, 8). The single Q fever vaccine approved for use in humans, Q-VAX^®^, is an inactivated whole cell vaccine based on phase I *C. burnetii* licensed for use only in Australia amongst high-risk individuals. The vaccine is protective against Q fever (9) but has been reported to cause side effects in previously exposed individuals, particularly at the site of injection (10, 11). Therefore, only unexposed individuals can be vaccinated, and pre-screening is required prior to vaccination using an intradermal skin test and serology to assess for any prior immunity against *C. burnetii*. To eliminate this requirement for pre-screening and the associated cost and time, a number of efforts seek to develop novel vaccines that protect against Q fever whilst having minimal side effects and thus eliminate the need for pre-screening (12–14). These efforts would benefit from a more complete understanding of the cellular components contributing to immune responses to *C. burnetii* (15).

Previous studies characterizing human cellular recall responses in the context of *C. burnetii* exposure have largely focused on cytokine release in response to inactivated or viable *C. burnetii* antigen preparations using whole blood, PBMCs or isolated dendritic cell populations from healthy and exposed, convalescent individuals, patients with chronic *C. burnetii* infection, and those suffering from Q fever fatigue syndrome (16–23). Flow cytometric analysis in humans has been restricted to *ex vivo* phenotypic analysis of circulating innate and adaptive immune cells in individuals with Q fever endocarditis (18, 19, 24, 25). The only studies that have analyzed *ex vivo* recall responses by individual peripheral immune cell populations to *C. burnetii* by cytometry have been conducted in mice pre-sensitized by infection or through vaccination (26, 27).

The goal of the current study was therefore to develop an expanded description of the cell populations contributing to long-term recall responses years after natural exposure to *C. burnetii*. To this end, we performed a comprehensive characterization of innate and adaptive *in vitro* recall responses six to ten years after initial infection in individuals naturally exposed to and convalescent from *C. burnetii* infection during the 2007-2010 Dutch Q fever outbreak. This cohort included four subgroups of individuals based on differing *C. burnetii-*specific IFNγ responses, serological status, and self-reported clinical symptoms during their initial infections. We assessed *in vitro* recall responses to heat-inactivated *C. burnetii* using a combination of bulk cytokine secretion as well as single cell phenotypic analysis by flow cytometry and mass cytometry. Both bulk cytokine release and single cell data showed that both adaptive and innate cellular compartments exhibit long-term increased responses following natural exposure to *C. burnetii*, which should prompt future studies to determine whether these innate responses also contribute to protection from infection by the pathogen.

## Materials and methods

### Ethics statement

The human study was reviewed and approved by the Medical Ethical Committee Brabant (Tilburg, Netherlands, NL51305.028.15) and all donors provided written informed consent.

### Human study cohort

The human study cohort has been previously described (28). In brief, Q-fever exposed individuals were recruited from a cohort characterized in a previous large Q fever study conducted in the village of Herpen, the Netherlands (29), which experienced a high incidence of *C. burnetii* infection during the 2007-2010 Q fever outbreak (30). In this previous study, 80% of the adult village population was screened in 2014 for evidence of adaptive immunity towards *C. burnetii*. Individuals were invited to participate in this current study following pre-selection based on clinical history and adaptive *C. burnetii*-specific immune responses determined in 2014. All study participants, including immunologically *C. burnetii*-naive controls, resided during and following the Q fever outbreak in the same area and were assigned to study groups based on clinical history and immunological assays performed at the time of study enrollment in 2015. Cellular immunity to *C. burnetii* was assessed with the CE-marked Q-Detect interferon-γ release assay (31) as described previously (28). To maximize the potential to detect *C. burnetii*-specific T cells in this cohort who were predominantly enrolled for epitope-screening (28), preference was given to donors with strong responses to whole heat-killed *C. burnetii* in the IGRA and without potentially confounding immune disorders. In total, 143 participants provided written informed consent. IGRA responses were re-assessed upon enrollment in October 2015, and serological status was additionally assessed by immunoblot. Volunteers were allocated to control Group 1 if they had no history of Q fever disease (29), were negative (32) by IGRA and by immunofluorescence assay (IFA) in spring 2014, and were again negative by IFA and immunoblot when enrolled in the previous study (28) in autumn 2015. The remaining volunteers that were positive by IGRA in spring 2014 were subdivided based on their serostatus (IFA in spring 2014 and immunoblot analysis in autumn 2015) and past Q fever disease (either registered (notified) in the national surveillance system, or self-reported) into Group 2 (seronegative, asymptomatic), Group 3 (seropositive, asymptomatic) and Group 5 (seropositive, symptomatic). A single volunteer fell into Group 4 (seronegative, symptomatic) and was not included in further analysis. At the time of enrolment, all volunteers were convalescent from their original exposure to *C. burnetii*, and none of the volunteers included in this study had known active *C. burnetii* infection or were diagnosed as suffering from acute or chronic Q fever.

All peripheral blood samples for this study were obtained between July 2016 and May 2017, i.e., six to ten years after initial exposure during the 2007-2010 Q fever outbreak in the Netherlands. A total of n=64 individuals contributed to one or more aspects of this immune profiling study (**Tables 1**, **2**).

**Table 1:** Human study subjects.

Group	N	Age in years (median, range)^1^	Females N (%)	*C. burnetii* IGRA status^1^	*C. burnetii* Sero-status (IFA)^1^	Previous symptomatic Q-fever episode^2^	*C. burnetii*-specific IFNγ response in pg/ml (median, range)^1,3^
**G1**	21	56 [45–76]	11 (52%)	Neg	Neg	No	3.8 [0-29.4]
**G2**	10	54 [43–68]	6 (60%)	Pos	Neg	No	75.8 [29–460]
**G3**	10	56 [44–66]	6 (60%)	Pos	Pos	No	399 [106–1045]
**G5**	23	50 [32–72]	15 (65%)	Pos	Pos	Yes	384 [64–1048]
**All**	**64**	**54 [32–76]**	**37 (59%)**				

^1^At inclusion into the study in October 2015.

^2^Either formally notified or self-reported.

^3^Q-detect IGRA ELISA, background subtracted values.

Bold values indicate combined values for all study participants.

**Table 2:** Subject numbers included in each immune profiling assessment.

	Group				
Profiling study	G1	G2	G3	G5	All
Cytokine secretion	20	10	5	20	55
Flow cytometry ^1^	17	0	0	19	36
Mass cytometry	9	8	10	9	36

^1^Two rounds of flow cytometry profiling were performed; n=3 subjects from G1 and n=1 subject from G5 were included in both rounds.

### Whole blood IFNγ release assay (Q-Detect™ IGRA)

The Q-Detect™ assay has previously been described in detail (31). In brief, whole lithium-heparin anti-coagulated blood was stimulated with *C. burnetii* antigen (heat killed Cb02629, a strain isolated during the Dutch Q fever outbreak, lot 14VRIM014 prepared by Wageningen Bioveterinary Research from a master cell bank using a cell-free culture method and quality controlled for protein concentration, functional TLR stimulation, and stimulation of IFNγ in samples from known Q fever-exposed individuals). Assays were performed in 96-well polypropylene plates (Greiner BioOne) by adding 180 µl blood to 20 µl *C. burnetii* antigen diluted in phenol red-free RPMI supplemented with Glutamax (2 mM), Gentamycin (5 µg/ml) and sodium pyruvate (1 mM, all ThermoFisher Scientific). A 1.5% (v/v, final concentration) solution of PHA-M (ThermoFisher Scientific) was added to separate wells for each sample as a positive control. Medium only was added to the negative control wells for each sample, results from which were used to correct for any background levels of IFNγ in the sample. All stimulations were performed in duplicate. After 22 ± 1 hours whole blood cultures were re-suspended. IFNγ concentrations were assessed in whole blood by ELISA using the IFNγ Pelipair protocol (Sanquin) with minor modifications. The upper detection limit of IGRA under these conditions is 1050 pg/ml. A subject was scored positive by IGRA if the *C. burnetii antigen* induced IFNγ production was ≥16 pg/ml above background and the ratio of the logarithmic value of background-subtracted *C. burnetii* antigen and PHA responses ((log[*C. burnetii*]-log[neg control])/(log[PHA]-log[neg control])) was ≥0.4.

#### Multiplex cytokine secretion analysis following whole blood stimulation

Whole lithium-heparin anti-coagulated blood was stimulated with *C. burnetii* antigen in 1.5 mL microtubes (Eppendorf) by adding 500 µL blood to 55 µL diluted C. *burnetii* antigen or medium only. After 21-23 hours, plasma supernatants from whole blood cultures were collected and frozen for later multiplex cytokineanalysis. Quantification of secreted IFNγ, IL-1β, IL-2, IL-10 and TNFα was conducted using a sandwich ELISA-based multi-spot electrochemiluminescence detection system (human Proinflammatory Panel 1 V-PLEX kit, Meso-Scale Discovery), following the manufacturer’s recommendations. Cytokine levels in plasma supernatants measured following *C. burnetii* antigen stimulation were background corrected before data analysis by subtraction of levels detected in parallel medium-only assays for each sample. Notably, IFNγ levels measured using this V-PLEX assay are approximately 20 times higher than by the Q-Detect clinical IGRA used to assess the cellular immune status for enrolment and group allocation (28). This is because calibrators were differently dose-assigned by the two assay manufacturers. Despite this difference, IFNγ levels measured in the same set of samples by V-PLEX and Q-Detect IGRA directly correlate (R = 0.87, p = 1.44x10^-9^).

#### Flow cytometry analysis following whole blood stimulation

Lithium-heparin anti-coagulated whole blood was stimulated for 24-25 hours with *C. burnetii* antigen in 1.5 mL microtubes (Eppendorf) by adding 500 µL blood to 55 µL diluted C. *burnetii* antigen or medium only. For the final 20 hours of stimulation, Brefeldin A (Sigma) was added to a final concentration of 5 µg/mL. Two tubes were stimulated per donor and condition. Following stimulation, the two replicates of whole blood cultures were pooled, lysed using 10 mL 1x red blood cell (RBC) lysis buffer (eBioscience) and washed prior to staining and flow cytometry analysis. Each sample was evenly divided into three wells of a 96-well v-bottom plate (Sarstedt), washed with PBS and incubated for 30 min at 4°C with 50 µL fixable viability dye eF780 (eBioscience) diluted in PBS. Cells were washed twice with staining buffer (PBS/0.5% BSA), and stained with 25 µL antibody cocktail diluted in staining buffer for 20 min at 4°C. All antibodies used for surface and intracellular labelling are listed in **Tables 3**, **4**. Cells were washed and re-suspended in 50 µL fixation/permeabilization buffer (eBioscience), incubated for 45 min at room temperature, and washed with 150 µL permeabilization buffer (eBioscience). For intracellular staining, cells were incubated for 45 min at room temperature with 25 µL antibody cocktail diluted in permeabilization buffer (eBioscience).

**Table 3:** Flow cytometry staining panels Round 1.

Panel	Target	Clone	Label	Source
Fixable viability dye			eF780	Biolegend
T-cell panel (surface)	CD3	UCHT1	BV510	Biolegend
	CD4	RPA-T4	AF488	Biolegend
	CD19^x^	HIB19	APC-eF780	eBioscience
T-cell panel (intracellular)	CD14^x^	63D3	APC-Fire750	Biolegend
	CD16^x^	3G8	APC-Fire750	Biolegend
	CD154	24-31	PE	Biolegend
	IFNγ	4S.B3	PE-Cy7	Biolegend
	TNFα	MAb11	PerCp	Biolegend
	CD137^#^	4B4-1	BV421	Biolegend
Monocyte panel A+B (surface)	CD66b	G10F5	FITC	Biolegend
	CD3^x^	UCHT1	APC-eF780	eBioscience
	CD19^x^	HIB19	APC-eF780	eBioscience
Monocyte panel A (intracellular)	CD14	M5E2	BV421	Biolegend
	CD16^$^	3G8	BV510	Biolegend
	TNFα	MAb11	PerCp	Biolegend
	IL-1β	JK1B-1	AF647	Biolegend
Monocyte panel B (intracellular)	CD14	M5E2	BV421	Biolegend
	CD16^$^	3G8	BV510	Biolegend
	IL-6	MQ2-13A5	PerCp-Cy5.5	Biolegend
	IL-10	JES3-9D7	AF647	Biolegend

x Markers were used as part of the dump channel to exclude non-viable cells and those not of interest in this panel.

^$^CD16 expression on monocytes was adversely affected by stimulation; disregarded for monocyte (subpopulation) gating.

^#^Poor staining result with no identifiable positive population; disregarded for analysis.

**Table 4:** Flow cytometry staining panels Round 2.

Panel	Target	Clone	Label	Source
Fixable viability dye			eF780	Biolegend
T-cell panel A+B (surface)^§^	CD3	UCHT1	BV510	Biolegend
	CD4	RPA-T4	AF488	Biolegend
	CD19^x^	HIB19	APC-eF780	eBioscience
T-cell panel A (intracellular)	CD14^x^	63D3	APC-Fire750	Biolegend
	CD16^x^	3G8	APC-Fire750	Biolegend
	TNFα	MAb11	PerCp	Biolegend
	IL-2	MQ1-17H12	PE	Biolegend
	T-bet^$^	4B10	BV421	Biolegend
T-cell panel B (intracellular)	CD14^x^	63D3	APC-Fire750	Biolegend
	CD16^x^	3G8	APC-Fire750	Biolegend
	CD154	24-31	PE	Biolegend
	IFNγ	4S.B3	BV421	Biolegend
	CD25^#^	M-A251	PECy7	Biolegend
	FOXP3^#^	PCH101	eF660	eBioscience

x Markers were used as part of the dump channel to exclude non-viable cells and those not of interest in this panel.

^+^Staining was adversely affected by stimulation and disregarded during gating.

^$^Poor staining with no clearly identifiable T-bet positive population and no differences between stimulated and un-stimulated blood.

Poor staining result with no identifiable CD25+FOXP3+ positive population; excluded from analysis.

^§^T cell panel A also included CD8 PE-Cy7 (clone RPA-T8); disregarded during gating since none of the other T cell panels included CD8.

Single stains for compensation were prepared using lysed unstimulated and stimulated whole blood cultures as appropriate. Cells were washed with permeabilization buffer and re-suspended in 200 µL PBS/1% paraformaldehyde. Samples were acquired on a Gallios flow cytometer (BeckmanCoulter) and a FACSCanto II (BD Biosciences). A minimum of 100,000 events per sample was acquired. Data analysis was performed with FlowJo v10 software, using a combination of manual, magnetic and tethered gating (**Supplementary Figures S1, S2**). Four intracellular markers (CD137, T-bet, FOXP3 and IL-10) had no clearly identifiable positive population and were hence excluded from analysis.

#### Mass cytometry analysis following PBMC stimulation

Mass cytometry analysis was conducted using stimulated PBMCs rather than whole blood, since initial experiments showed that processing of stimulated whole blood for mass cytometry resulted in cell clumping and massive cell loss during the multiple wash steps required to remove debris after RBC lysis, thereby compromising CyTOF analysis.

PBMCs were isolated from lithium-heparin anti-coagulated blood using Leukosep tubes prefilled with Ficoll (Greiner BioOne) according to the manufacturer’s recommendations. Prior to the final wash and counting, erythrocytes were lysed using 1X RBC lysis buffer (eBioscience). Freshly isolated PBMCs were stimulated for 18-20 h at 1x10^6^ cells per well in 96-well U-bottom plates (Corning) in a final volume of 100 µL complete RPMI (phenol red-free RPMI supplemented with 2 mM Glutamax, 5 µg/mL gentamycin, and 1 mM sodium pyruvate; all Thermo Fisher Scientific) with 10% fetal bovine serum (HyClone). For the final 14 hours of stimulation, Brefeldin A (Sigma) was added at a final concentration of 5 µg/mL. Stimulations for each donor were carried out with *C. burnetii* antigen or medium only in five replicate wells each. To accommodate the logistics of sample collection and processing, antigen stimulation and initial staining were performed in four separate runs of nine donors from different combination of two to three groups, for a total of thirty-six donors (Run 1: n=5 Group 3, n=4 Group 5; Run 2: n=3 Group 1, n=1 Group 2, n=5 Group 3; Run 3: n=6 Group 1, n=3 Group 2; Run 4: n=4 Group 2, n=5 Group 5).

Cisplatin staining, surface marker staining and barcoding of freshly isolated and stimulated PBMC samples were conducted at Innatoss in the Netherlands, while intracellular staining and mass cytometry analysis were conducted at MGH in the United States. Cryopreserved PBMCs from two donors with no history of Q fever disease were included as reference samples in each stimulation and staining run. Unstimulated cells from one donor were included in each staining run served as a control sample for surface antibody labelling. To establish a control sample for intracellular staining, freshly isolated PBMCs from a second donor were stimulated with a combination of *Staphylococcus aureus* enterotoxin B (final concentration 1 µg/mL, Sigma) and LPS (final concentration 100 ng/mL, eBioscience), cisplatin- and surface-labelled, barcoded, fixed, aliquoted and frozen. A single aliquot of this stimulated sample was included in each run as a control for intracellular staining.

Following stimulation, cells from the five replicate wells per donor and stimulation condition were pooled and washed four times with staining buffer (PBS/0.5% BSA) prior to surface and cisplatin staining. All washes and staining procedures were carried out in 1.5 mL microtubes (Eppendorf). For surface staining of markers sensitive to fixation during the barcoding procedure, cell pellets were resuspended in surface antibody cocktail 1 (anti-CD56 and anti-CD16, **Table 5**) diluted in staining buffer and cells were incubated for 60 min at 4°C. For the final 10 min of incubation, Cell-ID™ Cisplatin (Fluidigm; final concentration 2.5 μM) was added. Following staining, each sample was washed three times and counted. Up to 3x10^6^ cells per sample were used for barcoding using the Cell-ID™ 20-Plex Pd Barcoding Kit (Fluidigm). For barcoding, samples were first incubated in 400 μL Fix buffer and incubated for 10 min at room temperature, followed by two washes with Barcode Perm buffer (Fluidigm). Each sample was individually barcoded in a final volume of 120 μL barcode in Barcode Perm buffer for 30 min at room temperature. Following two washes with MaxPar cell staining buffer, each cell pellet was resuspended in 100 μL MaxPar cell staining buffer. All unstimulated and *C. burnetii* antigen-stimulated samples from n=9 donors and the unstimulated reference sample were then combined, pelleted and resuspended in 1 μL surface antibody cocktail 2 (**Table 5**) diluted in cell staining buffer, and incubated for 60 min at 4°C. Cells were then washed once more with cell staining buffer, resuspended in 150 μL 4% paraformaldehyde and incubated for 2 hours at room temperature to inactivate any potential live *C. burnetii* per requirements of the MGH Biosafety Committee (27). Samples were then frozen in a bulk-freezing container (CoolCell) and shipped on dry ice to MGH for intracellular staining and mass cytometry analysis.

**Table 5:** Mass cytometry staining panel.

Cocktail	Target	Clone	Label	Source
Antibody cocktail 1 (surface)	CD16	3G8	209Bi	Fluidigm Inc.
	CD56	NCAM16.2	162Dy	BWH
Antibody cocktail 2 (surface)	CD11c	Bu15	159Tb	BWH
	CD137 (4-1BB)	4B4-1	166Er	BWH
	CD14	M5E2	151Eu	Fluidigm Inc.
	CD184 (CXCR4)	12G5	173Yb	Fluidigm Inc.
	CD19^#^	HIB19	160Gd	BWH
	CD197 (CCR7)^#^	G043H7	167Er	Fluidigm Inc.
	CD20	2H7	148Nd	BWH
	CD206 (MMR)	15-Feb	145Nd	BWH
	CD27	O323	141Pr	BWH
	CD3	UCHT1	158Gd	BWH
	CD33	WM53	163Dy	BWH
	CD38	HIT2	172Yb	Fluidigm Inc.
	CD4	RPA T4	155Gd	BWH
	CD45	HI30	89Y	Fluidigm Inc.
	CD45RA^#^	HI100	153Eu	BWH
	CD45RO^#^	UCHL1	149Sm	BWH
	CD66b	G10F5	171Yb	BWH
	CD69	FN50	144Nd	Fluidigm Inc.
	CD8a	RPA-T8	146Nd	BWH
	HLA-DR	L243	143Nd	Fluidigm Inc.
Antibody cocktail 3 (intracellular)	CD154 (CD40L)	24-31	168Er	BWH
	CD70 (113-16)	113-16	150Nd	BWH
	FOXP3	PCH101	161Dy	BWH
	GATA3	TWAJ	170Er	Fluidigm Inc.
	IFNγ	4S.B3	165Ho	BWH
	IL-10	JES3-19F1	176Yb	BWH
	IL-17A	BL168	169Tm	BWH
	IL-1β	H1b-27	154Sm	BWH
	IL-2	MQ1-17H12	164Dy	BWH
	IL-4	8D4-8	147Sm	Fluidigm Inc.
	IL-6	MQ2-13A5	156Gd	BWH
	IL-8	BH0814	142Nd	BWH
	Tbet	4B10	175Lu	BWH
	TGFβ	TW4-2F8	174Yb	BWH
	TNFα	Mab11	152Sm	BWH

^#^Markers showed very large batch effects and could not be normalized; excluded from analysis.

At MGH, frozen samples were thawed and labelled with intracellular antibodies for CyTOF; each run was processed on a separate day. One frozen vial of barcoded sample pool and one frozen vial of the stimulated barcoded reference sample were thawed in a 37°C water bath, pelleted and resuspended in 1 mL of 1X eBioscience Fix/Perm buffer for 30 minutes. Cells were then washed twice in 1X perm buffer, resuspended in 500 µL of intracellular antibody cocktail (**Table 5**) and incubated for 30 minutes at 4°C. Stained cells were washed twice with 1X Perm buffer, resuspended in 150 μL of 4% paraformaldehyde and incubated for 10 minutes at room temperature. Post incubation, cells were pelleted, resuspended in cell staining buffer, and stored at 4°C overnight. The next day the cells were pelleted, resuspended in Cell-ID™ Intercalator Iridium (Fluidigm; final concentration 0.125 μM) and incubated at room temperature for 20 minutes. Cells were then washed twice in cell staining buffer, twice in nanopure water (purified using a Millipore Milli-Q system) and resuspended at 1 million cells per mL in nanopure water containing EQ calibration beads (Fluidigm). The data were acquired on a Helios Mass Cytometer (Fluidigm) at 400-500 events per second.

### Mass cytometry data analysis

Data were retrieved from the Helios Mass Cytometer into FCS files. EQ calibration beads were used to normalize all FCS files, to minimize any variation that occurred during data acquisition on the Helios Mass Cytometer. This normalization was performed using the Fluidigm software and sample data were debarcoded using the Fluidigm Debarcoding software. Post-processing, following identification of viable singlet CD45^+^CD66b^-^ mononuclear cells, four major immune populations (CD4 T cells, CD8 T cells, B cells and innate immune cells) were manually gated using FlowJo 10 (**Supplementary Figure S3**).

Several markers showed strong run-to-run or day-to-day staining variation. For manual gating of CD14^+^ monocytes and cytokine producing cells, gates were therefore set per run based on the run-specific staining control sample. Prior to unsupervised clustering analysis, marker expression levels had to be additionally normalized across the four runs. This normalization, based on representative concatenate samples (see below), was performed separately for each immune subpopulation using CytoNorm (33) to remove batch-specific variations. Of the 37 antibodies included in the CyTOF panel, four markers (CD45RO, CD45RA, CD197/CCR7, CD19) showed very large batch-to-batch variation outside the range for effective normalization and were excluded from unsupervised clustering analysis. The canonical markers used to identify the four main cell populations (CD45, CD66b, CD3, CD4, CD8, CD20) were not included into the normalization process. The following mass cytometry markers were normalized using CytoNorm: CD16, FOXP3, CD56, CD33, IL-2, CD137, CD154, CD14, IL-6, IFNγ, T-bet, IL-8, HLA-DR, CD69, CD206, CD70, CD27, IL-4, TNFα, IL-1β, CD11c, IL-17, GATA3, CD38, CXCR4, TGFβ, and IL-10. A representative normalization sample was created for each of the four runs by down-sampling each debarcoded sample and concatenating the data. The four representative concatenate samples created in this manner were then used as the normalization reference samples in CytoNorm, to aid normalization of the data across all 4 runs. Since normalization was performed separately for each immune subpopulation, normalized expression levels for a given marker cannot be compared across the major immune populations. To assess if the normalization was accurate, we compared the control unstimulated PBMC sample included in all four runs as a staining control, before and after normalization (**Supplementary Figures S4–S11**). UMAP dimension reduction was performed on the four control samples prior to normalization, and separately after normalization. Before normalization, the surface and intracellular marker data for this control sample from different runs behaved like different samples. Post-normalization the control sample from each run overlaid the others, indicating that they were properly normalized.

Following normalization, data for all samples from all runs were clustered for each major cell population separately. Clustering was performed using FlowSOM (34), which employs self-organizing maps to identify immune cell clusters. Four major parental cell populations were defined as follows: CD4 T cells (CD3^+^CD4^+^CD8^-^CD20^-^); CD8 T cells (CD3^+^CD4^-^CD8^+^CD20^-^); B cells (CD3^-^CD20^+^); innate cells (CD3^-^CD20^-^). The mass cytometry markers used to cluster CD4 T cells, CD8 T cells, B cells and innate cells are listed in **Table 6**. FlowSOM parameters were initially set to identify 900 clusters for each major cell population. These 900 were subsequently meta-clustered to identify 20 meta-clusters from each major cell population (C1-C20). Using the SpadVizR R package, the median intensity of each marker was plotted in each meta-cluster to enable the phenotypic definition of the cell population via parallel coordinate plots. For each donor, the frequencies of each meta-cluster within each parental population in both control and *C. burnetii* antigen-stimulated conditions were calculated as a percent of the total number of cells clustered from that donor for that cell population. Subsequently, background corrected frequencies for each meta-cluster were calculated to identify whether the respective meta-cluster increased or decreased upon stimulation with *C. burnetii* antigen.

**Table 6:** CyTOF markers used in FlowSOM clustering.

CD4 T Cell	CD8 T Cell	B Cell	Innate Cell
CD16	CD16	CD16	CD16
FOXP3	CD56	IL-2	CD56
IL-2	IL-2	CD137	CD33
CD137	CD137	CD154	IL-2
CD154	CD154	IL-6	CD137
IL-6	IL-6	IFNγ	CD154
IFNγ	IFNγ	T-bet	CD14
T-bet	T-bet	IL-8	IL-6
IL-8	IL-8	HLA-DR	IFNγ
HLA-DR	HLA-DR	CD69	T-bet
CD69	CD69	CD70	IL-8
CD70	CD70	CD27	HLA-DR
CD27	CD27	IL-4	CD69
IL-4	IL-4	TNFα	CD206
TNFα	TNFα	IL-1β	CD70
IL-1β	IL-1β	CD11c	CD27
CD11c	CD11c	IL-17	IL-4
IL-17	IL-17	TGFβ	TNFα
GATA3	GATA3	IL-10	IL-1β
CD38	CD38		CD11c
TGFβ	TGFβ		IL-17
IL-10	IL-10		TGFβ
			IL-10

### Statistical analysis

Statistical analysis was conducted based on the original group assignments (Groups 1, 2, 3, and 5), as well as combined group assignments, such as Group 2 + 3 + 5 (IGRA-positive). Statistical analysis was performed using GraphPad Prism 9. Spearman’s correlation analysis was conducted for all clusters and plasma cytokine secretion levels using R 4.1 and Hmisc. Heatmaps and dendrograms were created using Python 3.10 using Pandas, Pyplot and Seaborn. tSNEs were generated using Python 3.10 and Scikit-Learn.

## Results

### *In vitro C. burnetii*-specific whole blood cytokine release patterns reflect the serological and prior disease status of previously exposed individuals

The individuals included in this study were divided into four groups: Those with no apparent prior exposure based on the absence of humoral and cellular adaptive response (negative IFA and IGRA) and lack of clinical history (Group 1), and previously exposed individuals based on a measurable cellular response by IGRA. The presence of a cellular immune response to *C. burnetii* antigen suggests that the IGRA-positive individuals had experienced prior infection with *C. burnetii* even if the infection did not result in a clinical history of symptomatic Q fever. These IGRA-positive individuals were further subdivided based on serological and prior disease status into Group 2 (seronegative, asymptomatic), Group 3 (seropositive, asymptomatic) and Group 5 (seropositive, symptomatic). No individuals with known active *C. burnetii* infection, as either current acute or chronic Q fever, were included in the study.

**Figure 1:**
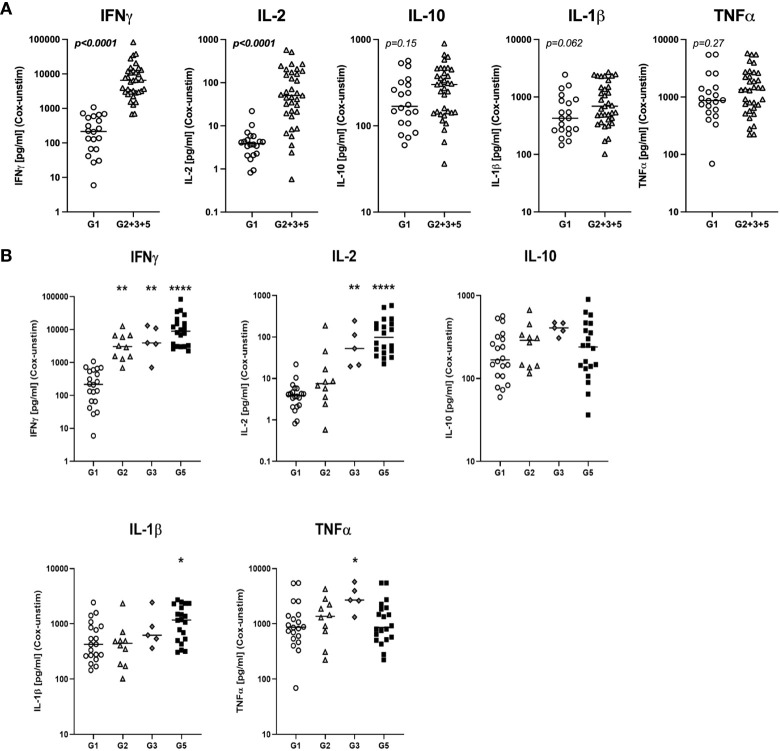
*C. burnetii*-specific whole blood cytokine release patterns. Multiplex cytokine secretion analysis was conducted on supernatants from whole blood stimulations for individuals without immunological evidence of prior exposure to *C. burnetii* (unexposed Group 1; n=20) and those with pre-existing humoral and/or cellular immunity (pre-exposed Groups 2, 3 and 5; n=10, n=5 and n=20, respectively). Background-corrected *C. burnetii-*specific cytokine data are shown for each individual. Data from Groups 2, 3 and 5 are combined in **(A)** and plotted separately in **(B)**. Lines indicate the median. Cytokine responses were compared using the Mann-Whitney U test in **(A)** and Kruskal-Wallis test followed by Dunn’s *post-hoc* multiple comparison test for nonparametric data in **(B)**. Asterisks are defined as follows: p > 0.05 (ns), p ≤ 0.05 (*), p ≤ 0.01 (**) and p ≤ 0.0001 (****). Background cytokine production levels in unstimulated samples across all groups were as follows (Median with interquartile range (IQR)): IFNγ 6.8 pg/mL (4.6-12.3), IL-2 0.20 pg/mL (0.20-0.20), IL-10 0.27 pg/mL (0.13-0.44), IL-1β 0.33 pg/mL (0.14-0.83), TNFα 2.4 pg/mL (1.7-3.6).

We first assessed whether the exposure status of individuals was associated with differential release of cytokines previously reported to be associated with re-stimulation responses to whole-cell *C. burnetii* antigen. We compared bulk cytokine responses as determined by multiplex V-PLEX assay following whole blood stimulation with heat-killed *C. burnetii* antigen in individuals with different states of prior exposure, clinical history, and serological status. Prior exposure (Groups 2, 3 and 5) was associated with significantly elevated release of IFNγ quantified by V-PLEX (p<0.0001; **Figure 1A**), consistent with the positive IGRA results used to define groups at study enrollment. IGRA responses (at enrollment) and IL-2 responses showed a strong correlation (Spearman Rho = 0.86, p= 4.5535E-09), and IGRA positivity was associated with significantly increased release of IL-2 (p>0.0001) compared to that observed in cells from unexposed individuals from group 1 (**Figure 1A**). In contrast, the innate cytokines IL-10, IL-1β and TNFα were released in response to *C. burnetii* antigen-stimulation by cells from both control (Group 1) and pre-exposed individuals (Groups 2, 3 and 5). Median levels of the three innate cytokines were elevated in pre-exposed individuals, although statistical analysis supported a trend toward differential levels only for IL-1β (p=0.062) (**Figure 1A**). No significant correlation was found between innate and adaptive cytokine responses, or amongst innate cytokines.

Dimensional reduction of whole blood cytokine release data from n=55 individuals by t-SNE followed by k means clustering of t-SNE embeddings identified two main clusters of study participants (**Supplementary Figure S12** **).** Cluster 1 comprised all control Group 1 individuals, a majority of Group 2 and n=1 donor from Group 3 who showed low IFNγ and IL-2 responses to stimulation with *C. burnetii*. Cluster 2 contained all remaining IGRA-positive individuals showing predominantly high IFNγ and IL-2 responses. TNFα, IL-1β and IL-10 responses to *C. burnetii* antigen did not correlate with the cluster groupings.

Further analysis of the clinical subgroups defined by clinical and immunological status showed that in contrast to the IFNγ responses used to define prior exposure, IL-2 responses were significantly elevated only in seropositive individuals (Group 3 and 5) regardless of symptoms during infection, while IL-1β release was elevated specifically in seropositive individuals who had convalesced from symptomatic infection (Group 5) (**Figure 1B**). Finally, TNFα release was significantly elevated only in the small group of pre-exposed individuals with past asymptomatic infection who were analyzed (n=5, Group 3).

Except for IFNγ, released cytokine levels in seronegative Group 2 individuals were indistinguishable from those in the control Group 1 (**Figure 1B**), and median IGRA responses in Group 2 were lower than in seropositive Group 3 and 5 individuals (**Table 1**). These lower cellular responses in individuals that fail to show sero-conversion has previously already been described in a much larger cohort study in *C. burnetii*-exposed individuals (31) as well as in individuals exposed to HIV (35–37), HBV (38), HCV (39), HSV-2 (40) and SARS-CoV-2 (41, 42).

Overall, of the bulk cytokine responses evaluated, IFNγ and IL-2 were the most informative for inferring prior exposure. Innate cytokines TNFα and IL-1β also showed some association with prior exposure by natural infection, consistent with previous results (43), but elevation was more marginal and restricted to specific subgroups of pre-exposed individuals.

### Flow cytometry analysis reveals stronger *C. burnetii-*specific *in vitro* recall responses by both T cells and monocytes from seropositive individuals convalescent from prior symptomatic infection

To explore the cellular source of these cytokines in *C. burnetii* antigen-stimulated whole blood cultures, we first performed flow cytometry analysis for a randomly selected set of n=36 donors from the two groups with the greatest difference in clinical and immunological status: IGRA-negative and IFA-negative individuals with no clinical history of Q fever (Group 1), and IGRA-positive and IFA-positive individuals with past symptomatic infection (Group 5). We focused on the assessment of *C. burnetii-*induced intracellular cytokine production by monocytes and CD4 T cells, effector cells that are known to promote killing of *C. burnetii* and clearance of infection (44, 45).

**Figure 2:**
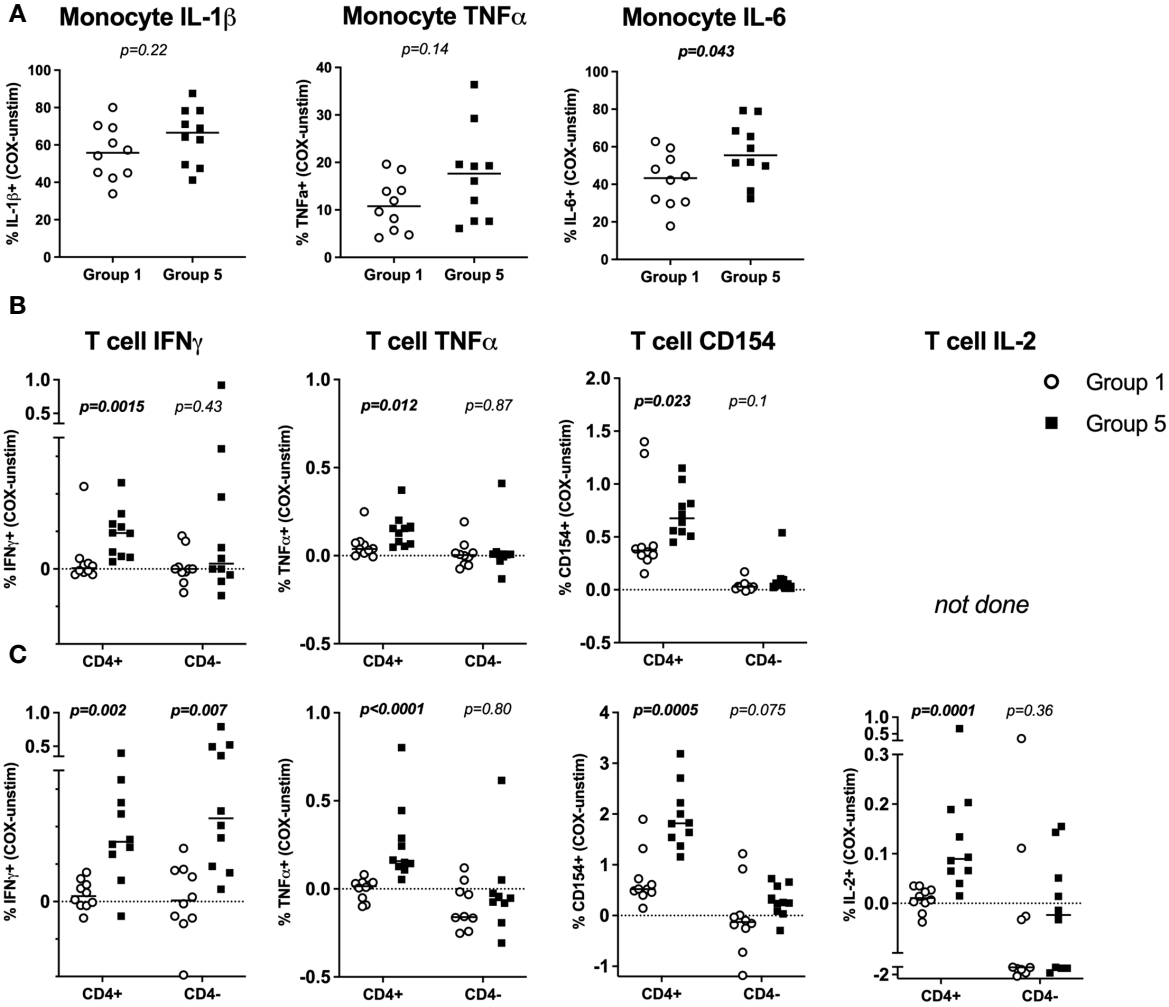
Flow cytometry profiles of *C. burnetii*-specific monocyte and T-cell cytokine production in stimulated whole blood. Cytokine production in whole blood following stimulation with *C. burnetii* was compared by flow cytometry between individuals without immunological evidence of prior exposure to *C. burnetii* (unexposed Group 1) and those with pre-existing humoral and cellular immunity as well as past symptomatic infection (exposed Group 5). **(A)** *C. burnetii*-specific cytokine responses by monocytes are depicted as the background corrected percentage of cytokine positive monocytes in unexposed Group 1 (n=10) and pre-exposed Group 5 (n=10) donors. Background proportions of cytokine-positive monocytes in unstimulated samples across the two groups were as follows (Median with IQR): IL-1β 0.53% (0.35-0.86), TNFα 0.14% (0.04-0.25), IL-6 0.43% (0.13-0.52). **(B, C)** *C. burnetii*-specific cytokine responses by T cells were assessed in two independent experimental rounds and using different T cell panels in a total of n=17 Group 1 and n=19 Group 5 donors. In each round, unexposed Group 1 (n=10) and exposed Group 5 (n=10) donors were included. Four donors were analyzed in both rounds. T cell cytokine responses are depicted as background corrected percentage of cytokine positive cells amongst CD4^+^ or CD4^-^ T cells. Background proportions of cytokine/activation marker positive T cells in unstimulated samples across the two groups was as follows in round 1 **(B)** (Median with IQR): CD4^+^ T cells IFNγ 0.01% (0.01-0.02), TNFα 0.04% (0.02-0.05), CD154 0.05% (0.03-0.07). CD4^-^ T cells IFNγ 0.02% (0-0.03), TNFα 0.03% (0.02-0.07), CD154 0.01% (0-0.02). In round 2 **(C)** the background values were (Median with IQR): CD4^+^ T cells IFNγ 0.07% (0.06-0.10), TNFα 0.09% (0.03-0.14), CD154 0.12% (0.06-0.17), IL-2 0.01% (0.01-0.03). CD4^-^ T cells IFNγ 0.11% (0.07-0.16), TNFα 0.45% (0.3-0.6), CD154 1.24% (0.30-3.44), IL-2 0.26% (0.09-0.99). Lines indicate the median. Cytokine responses between Groups 1 and 5 were compared using the Mann-Whitney U test for nonparametric data.

In *C. burnetii* antigen*-*stimulated whole blood, a high proportion of monocytes from both controls (Group 1) and IGRA-positive donors with a clinical history of Q Fever (Group 5) produced TNFα (median 10.8% and 17.6%), IL-1β (median 55.8% and 66.6%) and IL-6 (median 43.3% and 55.5%). Only the difference in IL-6^+^ monocytes between Groups 1 and 5 reached statistical significance (p = ≤0.05) (**Figure 2A**). However, there was a modest trend toward higher proportions of TNFα, IL-1β and IL-6 producing monocytes in pre-exposed Group 5 individuals.

T cell responses were assessed in two flow cytometry experiments, each including n=10 donors from each of Groups 1 and 5. Initially, a single T cell panel was used to assess IFNγ and TNFα production and the activation marker CD154 (**Figure 2B**). In a second round IL-2 production was also interrogated using a second T cell panel (**Figure 2C**). CD4 T cells consistently showed significantly higher proportions of IFNγ^+^, TNFα^+^ and CD154^+^ cells (**Figures 2B, C**) as well as IL-2^+^ cells (**Figure 2C**) in Group 5 individuals with clinical history of the disease compared to Group 1 controls. CD3^+^ CD4^-^ T cells, inferred as CD8 T cells, showed no significant difference in *C. burnetii*-induced TNFα or IL-2 production or CD154 expression between control (Group 1) and convalescent (Group 5) individuals (**Figures 2B, C**). However, amongst the Group 5 individuals assessed in round 1, a small number did show CD4^-^ IFNγ T cell responses of a similar magnitude as CD4 T cells (**Figure 2B**). Amongst the individuals in Group 5 assessed in round 2, this IFNγ response in CD4^-^ T cells was statistically significant and comparable to the response of CD4 T cells (**Figure 2C**). This suggests that CD8 T cells also contribute to recall IFNγ responses.

### Mass cytometry analysis identifies both adaptive and innate cell populations showing *in vitro* recall responses in individuals who were previously naturally exposed

The flow cytometry panels focused on assessing responses by monocytes and CD4 T cells. To characterize the immune cell subsets involved in recall responses to *C. burnetii* antigen more deeply and broadly, we utilized a highly multiplexed CyTOF panel in combination with manual gating and unsupervised clustering analysis. The CyTOF panel included additional lineage markers to investigate responses by CD8 T cells, B cells and innate immune cells, as well as a wider variety of activation markers and cytokines. For this CyTOF analysis we further expanded the selection of subjects: in addition to individuals from Group 1 (controls) and Group 5 (seropositive, symptomatic), we also included Group 2 (seronegative, asymptomatic) and Group 3 (seropositive, asymptomatic) individuals.

**Figure 3:**
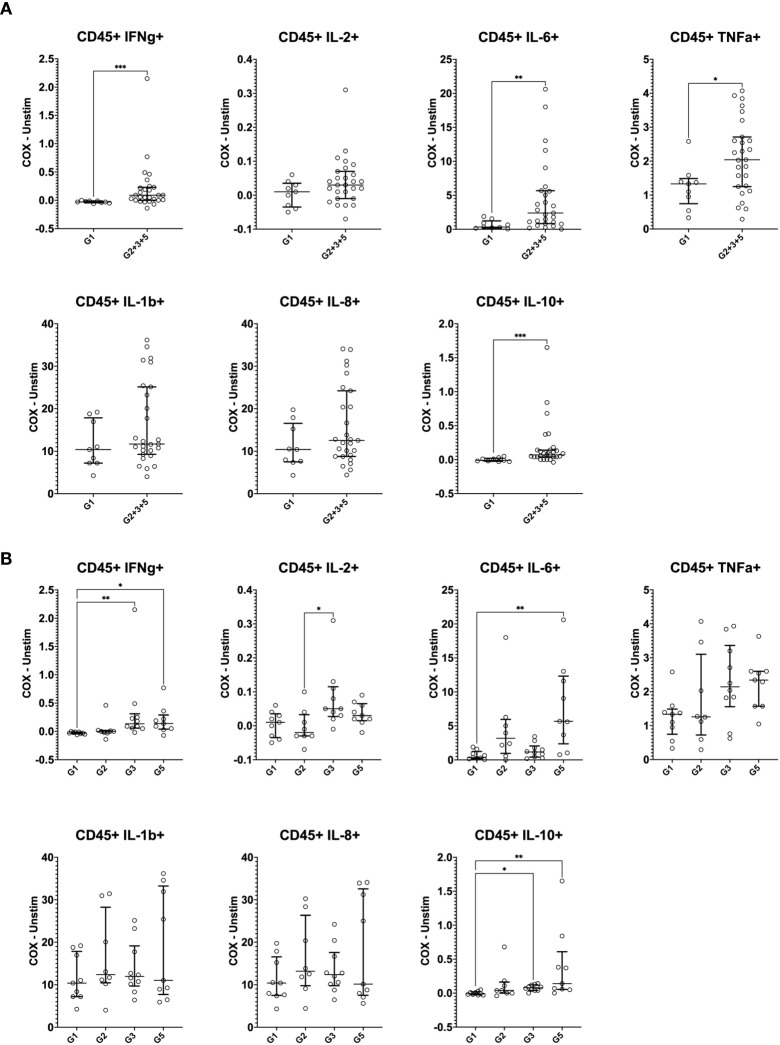
Mass cytometry profiles of *C. burnetii*-specific cytokine production in peripheral blood mononuclear cells. Cytokine production in PBMCs following stimulation with *C. burnetii* was assessed by mass cytometry. Scatterplots depict the background corrected percentage of each cytokine (IFNγ, IL-2, IL-6, TNFα, IL-1β, IL-8 and IL-10) in all CD45^+^CD66b^-^ mononuclear cells in: **(A)** unexposed Group 1 (n=9) and all pre-exposed individuals (n=27) donors (Group 2 + 3 + 5), **(B)** unexposed Group 1 (n=9), seronegative, asymptomatic Group 2 (n=8), seropositive, asymptomatic Group 3 (n=10) and seropositive, symptomatic Group 5 (n=9). Lines indicate the median with interquartile range. Cytokine responses were compared using Mann-Whitney U test in **(A)** and Kruskal-Wallis test followed by Dunn’s *post-hoc* multiple comparison test for nonparametric data in **(B)**. Asterisks are defined as follows: p > 0.05 (ns), p ≤ 0.05 (*), p ≤ 0.01 (**) and p ≤ 0.001 (***).

Cytokine responses were initially quantified in total CD45^+^CD66b^-^ mononuclear cells by manual gating. In *C. burnetii* antigen*-*stimulated PBMCs, the proportions of cells showing *C. burnetii*-specific production of IFNγ, IL-6, IL-10 and TNFα were significantly higher in pre-exposed IGRA-positive individuals compared to Group 1 controls (**Figure 3A**), consistent with IGRA responses at study enrollment (**Table 1**). The median increase with pre-exposure in cells producing these cytokines was two-fold or less, except for the proportion of IL-6^+^ cells which increased nearly six-fold. In contrast, the proportion of cells producing the innate cytokines IL-1β and IL-8 in response to *C. burnetii*-stimulation was comparable across the two groups and thus independent of prior exposure status. IFNγ levels were specifically elevated in seropositive Group 3 and 5 individuals, and the same was true for IL-10 and TNFα (**Figure 3B**). Although the proportion of IL-2-producing cells was not significantly different between IGRA-negative Group 1 controls and all IGRA-positive donors (Group 2 + 3 + 5) (**Figure 3A**), seropositive Group 3 and 5 individuals showed clearly higher IL-2 responses compared to Groups 1 and 2 (**Figure 3B**), with the difference between Group 2 and 3 individuals reaching statistical significance. *C. burnetii*-specific IL-4 responses were not detectable, and IL-17 responses were only detectable in a very minor proportion of cells (<0.05%) and showed no differences between IGRA-negative and IGRA-positive groups (data not shown).

**Figure 4:**
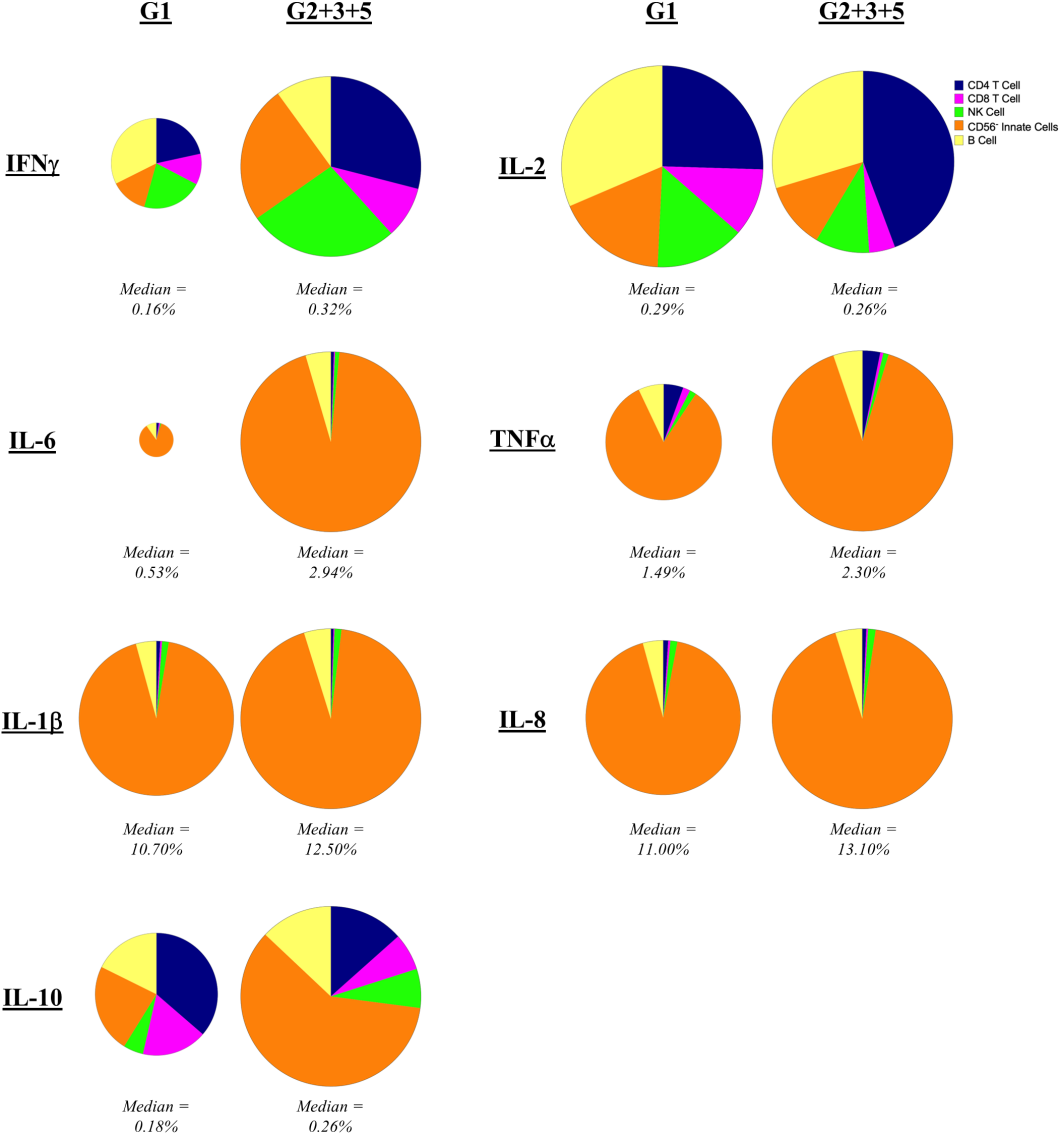
Contribution of mononuclear cell populations to *C. burnetii*-specific cytokine production. The contribution of CD45^+^CD66b^-^ cell populations in PBMCs to cytokine production following stimulation with *C. burnetii* was assessed by mass cytometry. Pie charts indicate the median percent contribution of each cell type (CD4 T cells, CD8 T cells, B cells, NK cells and CD56^-^ innate cells, to the frequency of cytokine-positive (IFNγ, IL-2, IL-6, TNFα, IL-1β, IL-8 or IL-10) cells in *C. burnetii*-stimulated samples (no background correction) in unexposed Group 1 (n=9) and all pre-exposed individuals (n=27) donors. The size of the pie chart for the pre-exposed donors (Group 2 + 3 + 5) is kept constant across panels. The pie chart of the unexposed group is scaled separately for each cytokine relative to the pre-exposed group, to reflect the difference in median frequency of cytokine positive CD45^+^ mononuclear cells. The median frequency of cytokine positive CD45^+^ mononuclear cells in *C. burnetii* stimulated samples for each group is displayed below each pie chart.

To assess the cellular source of these cytokines, we separately analyzed CD45^+^ mononuclear cells positive for each cytokine for individuals who were either IGRA-negative (Group 1) to IGRA-positive (Group 2 + 3 + 5) (**Figure 4**). IFNγ was broadly produced by all immune cell types evaluated, not solely by CD4 and CD8 T cells, regardless of the status of prior exposure. However, IFNγ expression by CD4 T cells (p = 0.029) and CD56^-^ innate cells (presumably monocytes and dendritic cells, p = 0.005) increased with prior exposure (higher proportion in Group 2 + 3 + 5 compared to Group 1). The primary sources of IL-2 were CD4 T cells and B cells. While the contribution of CD4 T cells to IL-2 production was slightly increased with prior exposure though this did not reach statistical significance. IL-6, TNFα, IL-1β and IL-8 were produced largely by CD56^-^ innate cells (presumably monocytes and dendritic cells), consistent with the canonical innate functions of these cytokines, with no difference in this pattern regardless of pre-exposure. CD4 T cells had the greatest contribution to IL-10 production in unexposed individuals (Group 1), but CD56^-^ innate cells became the predominant source in individuals with prior exposure (higher proportion in Group 2 + 3 + 5 compared to Group 1, p = 0.005).

**Figure 5:**
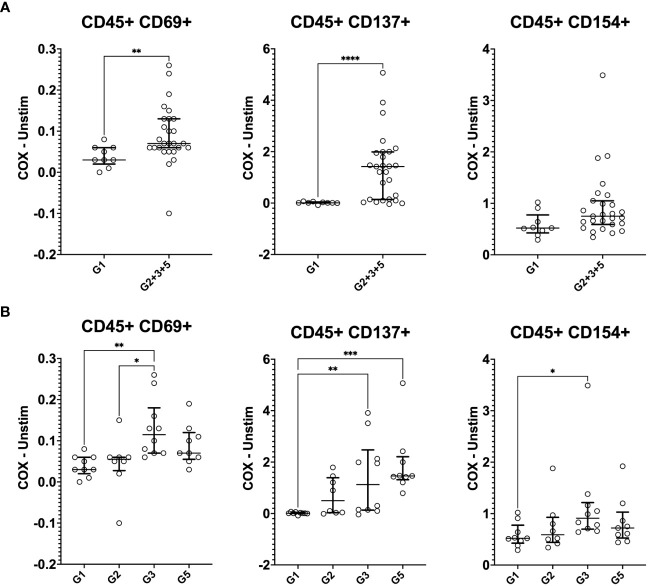
Mass cytometry profiles of *C. burnetii*-specific activation in stimulated peripheral blood mononuclear cells. Activation of CD45^+^CD66b^-^ cells following PBMC stimulation with *C. burnetii* was assessed by mass cytometry. Scatterplots depict the background corrected percentage for each activation marker (CD69, CD137 and CD154) in: **(A)** unexposed Group 1 (n=9) and all exposed individuals (n=27) donors (Group 2 + 3 + 5), **(B)** unexposed Group 1 (n=9), seronegative, asymptomatic Group 2 (n=8), seropositive, asymptomatic Group 3 (n=10) and seropositive, symptomatic Group 5 (n=9). Lines indicate the median with interquartile range. Activation responses were compared using the Mann-Whitney U test in **(A)** and Kruskal-Wallis test followed by Dunn’s *post-hoc* multiple comparison test for nonparametric data in **(B)**. Asterisks are defined as follows: p > 0.05 (ns), p ≤ 0.05 (*), p ≤ 0.01 (**), p ≤ 0.001 (***) and p ≤ 0.0001 (****).

In addition to cytokine production, IGRA-positive individuals also showed enhanced levels of activation markers expression in response to *C. burnetii* antigen stimulation of PBMCs, which was significant for CD69 and CD137 (**Figure 5A**). A more granular analysis of the different exposure groups showed that relative to the control Group 1, *C. burnetii*-induced CD69 and CD154 expression were significantly elevated in seropositive Group 3 individuals only, while the proportion of cells expressing the activation marker CD137 was significantly increased in both seropositive Groups 3 and 5 (**Figure 5B**).

**Figure 6:**
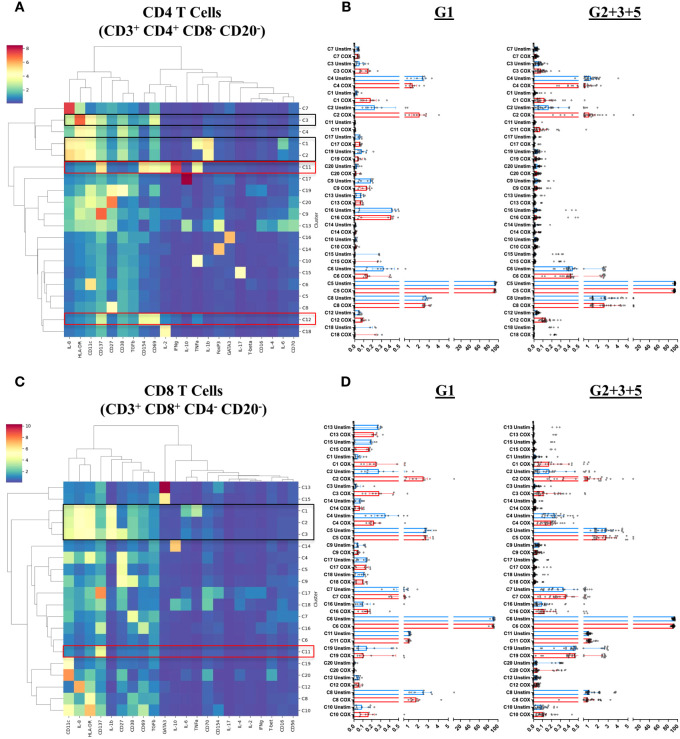
T cell subpopulations characterized by mass cytometry in peripheral blood mononuclear cells. Immune cell meta-clusters were identified through FlowSOM-based clustering of mass cytometry labeled samples. The phenotypic profile of each meta-cluster, in **(A)** CD4 T cells and **(C)** CD8 T cells, is depicted in heatmaps. The color gradient on the heatmaps indicates the mean metal intensity for each marker. Dendrograms were generated through hierarchical clustering using Ward linkages. The scatterplots on the right of each heatmap indicate the frequency (abundance) each meta cluster in **(B)** CD4 T cells and **(D)** CD8 T cells in each donor in unstimulated (blue) and *C. burnetii*-stimulated (red) samples. Lines indicate the median with interquartile range. Black boxes in heatmaps **(A)** and **(C)** highlight meta-clusters showing increased absolute abundance upon *C. burnetii* stimulation; red boxes highlight meta-clusters showing higher *C. burnetii*-specific relative abundance (background-corrected) in all pre-exposed individuals (Group 2 + 3 + 5) compared to Group 1.

Finally, we addressed the question of which specific cellular phenotypes characterize the innate and adaptive recall responses induced by *C. burnetii* antigen stimulation of PBMCs from pre-exposed individuals. To this end, FlowSOM clustering (34) identified 20 sub-populations (meta-clusters C1-C20) within each of the four major populations (CD4 T cells, CD8 T cells, B cells and Innate cells) amongst CD45^+^CD66b^-^ cells (**Figures 6A, C** **,** **7A, C**). We then assessed whether meta-cluster abundance was increased upon stimulation with *C. burnetii* antigen (indicating *C. burnetii*-specific responses, **Figures 6B, D** **,** **7B, C**) and whether the specific responses (assessed as background-corrected abundances) were higher in IGRA-positive (Group 2 + 3 + 5) individuals compared to Group 1 controls, and thus linked to prior exposure (**Figure 8**).

**Figure 7:**
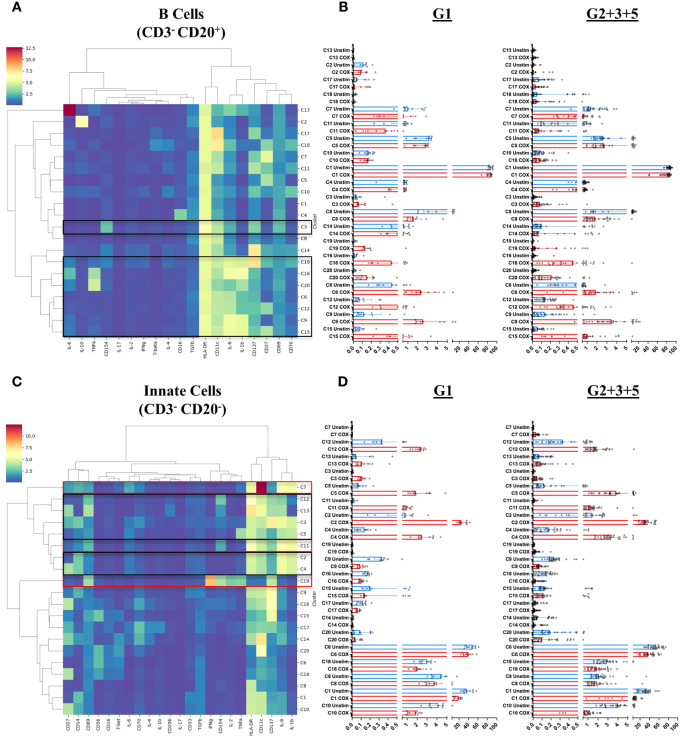
B cell and innate cell subpopulations characterized by mass cytometry in peripheral blood mononuclear cells. Immune cell meta-clusters were identified through FlowSOM based clustering of mass cytometry labeled samples. The phenotypic profile of each meta-cluster, in **(A)** CD3^-^CD20^+^ B cells and **(C)** CD3^-^CD20^-^ innate cells, is depicted in heatmaps. The color gradient on the heatmaps indicates the mean metal intensity for each marker. Dendrograms were generated through hierarchical clustering using Ward linkages. The scatterplots on the right of each heatmap indicate the frequency (abundance) each meta cluster in **(B)** B Cells and **(D)** Innate cells in each donor in unstimulated (blue) and *C. burnetii*-stimulated (red) samples. Lines indicate the median with interquartile range. Black boxes in heatmaps **(A)** and **(C)** highlight meta-clusters showing increased absolute abundance upon *C. burnetii* stimulation; red boxes highlight meta-clusters showing higher *C. burnetii*-specific relative abundance (background-corrected) in all pre-exposed individuals (Group 2 + 3 + 5) compared to Group 1.

**Figure 8:**
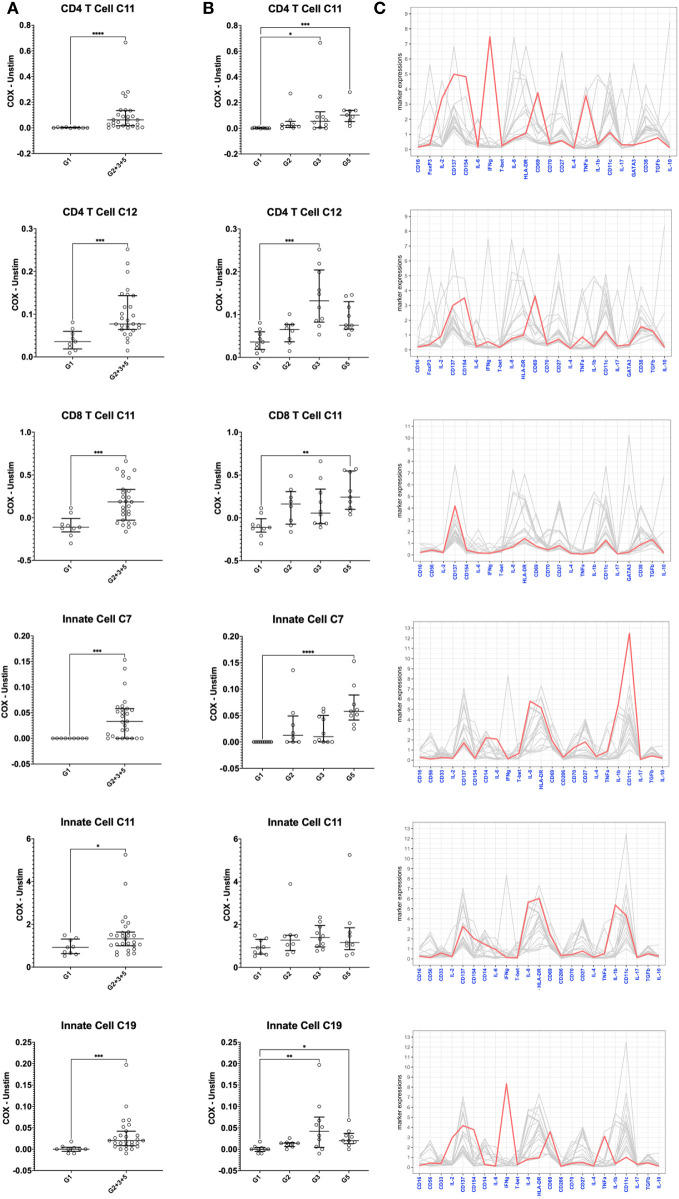
Mass cytometry profiles of *C. burnetii*-specific cellular responses in peripheral blood mononuclear cells. Cellular responses following stimulation with *C. burnetii* were assessed by mass cytometry. Scatterplots depict the background corrected percentage of relevant immune cell subpopulations (meta-clusters) in: **(A)** unexposed Group 1 (n=9) and all exposed individuals (n=27) donors (Group 2 + 3 + 5), **(B)** unexposed Group 1 (n=9), seronegative, asymptomatic Group 2 (n=8), seropositive, asymptomatic Group 3 (n=10) and seropositive, symptomatic Group 5 (n=9). Lines indicate the median with interquartile range. Activation responses were compared using the Mann-Whitney U test in **(A)** and Kruskal-Wallis test followed by Dunn’s *post-hoc* multiple comparison test for nonparametric data in **(B)**. Asterisks are defined as follows: p > 0.05 (ns), p ≤ 0.05 (*), p ≤ 0.01 (**), p ≤ 0.001 (***) and p ≤ 0.0001 (****). **(C)** Parallel coordinate plots depicting the mean intensity of each marker in the different clusters. Y axis indicates mean metal intensity. Line colored in red depicts the mean metal intensity of markers in that cluster, while lines in gray are depicting the mean metal intensity of markers in all other clusters.

Several meta-clusters amongst both CD4 and CD8 T cells showed enhanced abundance upon stimulation with *C. burnetii* antigen. CD4 and CD8 meta-clusters exhibiting *C. burnetii*-specific production of the innate cytokines IL-1β and IL-8 alone (CD4 C2, CD8 C2 and CD8 C3) or in combination with IL-6 and TNFα (CD4 C1 and CD8 C1) did not increase with prior exposure status, or were only found in unexposed individuals (CD4 C3 IL-8^+^) (**Figure 6**). In contrast, the relative abundances of two CD4 T cell meta-clusters producing adaptive cytokines were significantly higher in pre-exposed individuals: CD4 C11 (CD137^+^CD154^+^CD69^+^IFNγ^+^IL-2^+^TNFα^+^) and C12 (CD137^+^CD154^+^CD69^+^) (**Figure 8**), consistent with the elevated production of these cytokines by CD4 T cells in Group 5 individuals, as determined by flow cytometry (**Figure 2**). Amongst CD8 T cells, only C11 (CD137^+^) showed a higher relative abundance in pre-exposed individuals. However, this CD8 meta-cluster showed no effector cytokine production, and the difference in abundance between *C. burnetii* antigen-stimulated versus unstimulated samples was very minimal, especially in view of the abundance of this meta-cluster in unstimulated PBMCs (**Figure 6B**).

In B cells, eight of the 20 meta-clusters (C3, C19, C16, C20, C6, C12, C9 and C15) showed an increased abundance upon stimulation with *C. burnetii* antigen in all individuals. The majority of these *C. burnetii* antigen-stimulation associated B cell meta-clusters was again characterized by production of the innate cytokines IL-1β and IL-8 alone (C19, C6, C12, C9, C15) or in combination with TNFα (C20) or TNFα and IL-6 (C16), and for all relative abundance was unrelated to prior exposure. The same was true for the rare B cell meta-cluster C3 with an activated phenotype but lacking cytokine production (**Figures 7A, B**). No B cell cluster showed evidence for pre-exposure related *C. burnetii*-specific responses.

Amongst innate cells, there was again an increased abundance upon stimulation with *C. burnetii* antigen in all individuals, regardless of prior exposure, of meta-clusters showing production of IL-8 only (C12 and C13; likely dendritic cells that lack CD14 and CD56 expression and express high levels of HLA-DR and CD11c) or IL-1β and IL-8 (C3, C5, C2 and C4; CD14^+^ monocytes) (**Figures 7C, D**). Three populations of innate cells, however, showed a significantly higher relative abundance in pre-exposed individuals: the innate meta-clusters C7 (CD11c^high^CD14^+^HLA-DR^+^IL-1β^+^IL-6^+^IL-8^+^) and C11 (CD11c^int^CD14^+^HLA-DR^+^CD69^+^CD137^int^CD154^int^IL-1β^+^IL-6^low^IL-8^+^), both likely representing monocytes, and a meta-cluster lacking expression of any canonical markers, C19 (CD69^+^CD137^int^CD154^+^IFNγ^+^IL-2^+^TNFα^+^) (**Figure 8**).

When we further focused on the separate subgroups of IGRA-positive individuals, CD4 and CD8 T cell clusters C11 and innate cell cluster C7 all were significantly increased in Group 5 (seropositive, convalescent from symptomatic infection), while CD4 T cell cluster C12 and innate cell cluster C19 were highest in Group 3 individuals (seropositive, asymptomatic) (**Figure 8**). Background-corrected frequencies for all T cell and innate cell meta-clusters correlated with IFNγ levels released following stimulation of whole blood, and CD4 T cell cluster C11 and C12 frequencies additionally correlated with IL-2 levels (**Supplementary Table 1**).

## Discussion

In this study we examined the immune cell subpopulations contributing to long-term *C. burnetii*-specific recall responses approximately ten years post exposure in individuals who contracted Q fever during the Dutch Q fever outbreak of 2007 to 2010. These responses were quantified using IGRA, multiplex cytokine secretion assay, flow cytometry analysis of CD4 T cell and monocyte responses, and mass cytometry profiling of diverse cell populations. In general, we found that prior exposure to *C. burnetii* was associated with long-term increased recall responses in not only adaptive but also innate immune cell compartments.

Recall responses in humans have previously been analyzed solely by means of bulk cytokine secretion from peripheral immune cells (16, 17, 19, 21–23). To perform a broader and more in-depth analysis of the peripheral immune system we utilized both classical flow cytometry and mass cytometry (CyTOF), which uses heavy metal ion-labeled antibodies and time-of-flight mass spectrometry. This technology permits a much greater number of markers to be probed in parallel in the same sample and in combination with unsupervised clustering analysis facilitates the identification of novel immune subsets and signatures (46). For instance, CyTOF has been applied to analyses following influenza vaccination (47), during acute Zika virus (48) or SARS-CoV2 infection (49, 50) as well as following experimental human *Streptococcus pneumoniae* challenge (51).

Our study had several limitations. These included the relatively small number of individuals enrolled for the different subgroups in CyTOF analysis, resulting in limited power to detect statistically significant differences. The division of seropositive individuals into past symptomatic and asymptomatic infection was further based on self-reported data collected in 2015, i.e. five to eight years after the epidemic and may therefore not be fully accurate. Finally, we faced a few technical challenges. In the flow cytometry T cell panels, several markers performed poorly with no clearly identifiable populations (CD137, FOXP3, T-bet, IL-10). CyTOF analysis had to be conducted on PBMCs rather than whole blood, and the limitation of barcoding to 20 samples per CyTOF run made it necessary to assess individuals in four different runs on different days. The batch-to-batch variation between the four rounds precluded normalization of some markers which hence were not carried forward for downstream analysis. As these included the markers to distinguish memory and naïve T cells (CD45RO, CD45RA and CCR7), this precluded analysis of these subsets. Despite these limitations, the results of this study both confirm prior observations and highlight novel aspects of the immune responses to *C. burnetii* that merit future investigation.

The innate and adaptive immune response to *C. burnetii* infection is multi-faceted (52–55). Innate immune responses are the first line of defense during acute infection and direct the induction of adaptive immune responses. However, *C. burnetii* invades and replicates in monocytes and macrophages - key cellular components at the interface of the innate and adaptive immune response - and deploys a range of immune evasion mechanisms to avoid intracellular killing in these innate immune cells (56). Being an intracellular pathogen, the clearance of *C. burnetii* infection largely relies on cellular adaptive immune responses. While antibodies are required to control tissue damage in murine models, they are not sufficient to control infection. Instead, in these animal models T cell responses are critical to controlling early infection, mediating bacterial clearance and conveying vaccination-induced protection (52–55, 57, 58). In addition, there appears to be a greater role for major histocompatibility complex (MHC) class I-restricted CD8 T cell responses during primary infection in murine models (52, 57). For protection from secondary infection, in contrast, there is a greater role for MHC class II-restricted CD4 T cells as well as MHC II-restricted but CD4-independent mechanisms (58). Notably, IFNγ is required for the clearance of primary *C. burnetii* infection (52), but appears to be less critical for clearance during secondary infection in mice (58).

T cells elicit recall responses through IFNγ production (59), which results in *C. burnetii* killing in infected monocytes in a TNFα-dependent manner (44, 45). IFNγ secretion in response to stimulation with *C. burnetii* antigen is known to be enhanced in those having experienced prior infection (31, 59). We detected increased production not only of IFNγ but also of IL-2, IL-6, IL-10 and TNFα following *C. burnetii* antigen stimulation of peripheral immune cells isolated from individuals with prior exposure to *C. burnetii*. Flow cytometry and CyTOF both revealed that CD4 T cells were the most consistent contributors to IFNγ and IL-2 recall responses, while monocytes were the predominant source of IL-6, IL-8, IL-10 and TNFα. Additionally, there were some previously exposed donors who also showed elevated IFNγ response by flow cytometry in CD4-negative T cells (i.e. likely CD8 T cells). In our study, using CyTOF we also identified a population of *C. burnetii*-specific CD4 T cells (CD137^+^CD154^+^CD69^+^IFNγ^+^IL-2^+^TNFα^+^) that was significantly increased in IGRA-positive individuals, particularly those who had exhibited clinical symptoms and remained seropositive. Co-production of these cytokines by specific cell subsets is consistent with data from a prior study looking at bulk cytokine responses to *C. burnetii* in individuals with prior exposure to *C. burnetii* (43), which showed that individuals with prior exposure and high IFNγ production also produce high amounts of IL-2. Notably, the CD137^+^CD154^+^ CD69^+^IFNγ^+^IL2^+^TNFα^+^ CD4 T cell cluster C11 and the corresponding lineage-negative innate cell cluster C19 were the only two cell populations showing clear IFNγ production based on the expression-normalized CyTOF data used for unsupervised clustering. The data normalization likely reduced the ability to detect cell populations with lower IFNγ production, such as NK cells and CD8 T cells, which were clearly identifiable by manual gating of CyTOF data or flow cytometry data (for CD4^-^ T cells). Additionally, at least part of the IFNγ responses observed by flow cytometry in the CD4^-^ T cell compartment might originate from unconventional γδ T cells, which largely lack CD4 and CD8 expression. Vγ9 Vδ2 T cells have been shown to be activated and expanded during acute human *C. burnetii* infection (60), likely by *C. burnetii*-derived phosphoantigens since Vγ9 Vδ2 T cell activation requires expression of butyrophilin molecules BTN2A and BTN3A on monocytes (61). Since IFNγ is a key cytokine produced by Vγ9 Vδ2 T cells, and these cells have been shown also display the same hallmark ability as adaptive T cells to respond more strongly upon re-exposure (62), it is conceivable that they also contribute to recall responses against *C. burnetii*.

A key finding of our study is that beyond recall responses by adaptive T cells, we also found several cell populations within the innate compartment that showed increased responses to *C. burnetii* antigen in pre-exposed (IGRA-positive and seropositive) individuals compared to controls with no evidence of adaptive immunity to *C. burnetii* (**Figures 7C, D**, **8**). This was particularly evident in individuals with past symptomatic infection (designated Group 5): *C. burnetii* antigen-stimulated whole blood from these individuals showed elevated bulk release of IL-1β as well as increased IL-6 production by monocytes as detected by flow cytometry. We also observed a trend for increased whole blood release of IL-6 and TNFα, which did not reach significance. This is likely due to the small number of individuals assessed in this analysis, since in a previously published study with considerable larger groups from the same village cohort, release of both IL-6 and TNFα from *C. burnetii* antigen-stimulated whole blood was significantly higher in IGRA-positive individuals compared to controls (43). CyTOF analysis of *C. burnetii* antigen-stimulated PBMCs revealed an increased proportion of two innate (CD3^-^CD20^-^) clusters in individuals with prior exposure. Innate cluster C7 with the phenotype CD11c^high^CD14^+^HLA-DR^+^IL-1β^+^IL-6^+^IL-8^+^ was particularly strongly increased in a subgroup of pre-exposed, past symptomatic individuals (Group 5), while C11 (CD11c^int^CD14^+^HLA-DR^+^CD69^+^CD137^int^CD154^int^IL-1β^+^IL-6^low^IL-8^+^) was increased in IGRA-positive (combined Group 2 + 3 + 5) donors.

Both innate clusters associated with prior exposure likely represent CD14^+^ monocytes, and we have previously already hypothesized, based solely on cytokine release data, that increased innate cytokine responses after *in vivo* exposure to viable *C. burnetii* might be due to trained immunity of myeloid cells such as has been described for other pathogens (63). This new cytometry dataset underscores this hypothesis, although it clearly requires further investigation, including experiments evaluating the epigenetic status of monocytes in *C. burnetii*-exposed individuals. Remarkably, these increased monocyte responses were evident six to ten years after primary exposure to *C. burnetii*, a much longer timeframe than the up to one year typically attributed to trained immunity based on the short lifespan of innate immune cells (64). On the other hand, trained immunity following BCG vaccination has been shown to last for up to five years, which may be partially attributed to the reprograming of hematopoietic progenitor cells (65, 66). Conceivably the long duration of enhanced innate re-call responses in our study might have also been facilitated by (asymptomatic) re-exposure. Indeed, elevated innate responses were most evident in Group 3 and 5 individuals, also showing the strongest adaptive responses. This also raises the question whether these stronger adaptive immune responses in seropositive individuals might have positively influenced training of innate responses. However, the contribution of adaptive responses to trained immunity is controversial, since IFNγ has been shown to both support and hamper the induction of trained immunity (64). Moreover, while stronger adaptive responses in Groups 3 and 5 could theoretically be a result of re-exposure, this in unlikely the case for all individuals in these cohorts since no large outbreaks have been reported after 2010. Of note, trained innate immunity is generally a property of live attenuated whole cell vaccines (67) and indeed we found no evidence for enhanced innate responses following vaccination with the killed whole cell vaccine Q-VAX (43). If such responses indeed are relevant to protection from or resolving future infection, approaches should be considered to combine future *C. burnetii* vaccines with amplifiers of trained immunity, such as BCG (67).

Another cell population that showed an increased abundance in *C. burnetii* antigen-stimulated cultures specifically for IGRA-positive previously exposed individuals was the presumed innate cluster C19 (CD69^+^CD137^mid^CD154^+^IFNγ^+^IL-2^+^TNFα^+^). Given the complete lack of lineage marker expression, one possible interpretation of this phenotype is that these cells constitute innate lymphoid cells (ILCs) of ILC1 or ILC3 polarization. IFNγ and TNFα production by these two ILC subsets has been shown to play an important role for the early host defense against a wide range of intracellular pathogens, as reviewed recently (68). Moreover, akin to adaptive lymphocytes, there is accumulating evidence that immunological memory is also a property of innate lymphoid cells and contributes to long-term protection also after vaccination (69). This is likely due to epigenetic modifications, as originally shown for ‘trained immunity’ in monocytes and recently demonstrated for ILC2 cells in asthma (70). Whether or not ILC1 or ILC3 cells indeed contribute to recall responses to *C. burnetii* requires further work beyond this study, using dedicated flow cytometry or mass cytometry panels (71, 72).

In conclusion, our cytometry data set profiling cellular recall responses in a cohort of individuals up to a decade after natural exposure to *C. burnetii* shows that CD4 T cells are the major driver of previously reported *C. burnetii*-specific production of IFNγ and IL-2. In addition, we find evidence that an innate population possibly resembling ILCs also contributes to this Th1-type re-call response. Finally, our data show that although numerous cell populations in the innate and adaptive compartments produce innate cytokines upon *C. burnetii* antigen stimulation regardless of prior exposure, the release of innate cytokines IL-1β, IL-6 and IL-8 and the proportion of two distinct monocyte populations expressing these cytokines in response to *C. burnetii* was specifically elevated in previously exposed individuals, indicative of trained innate immunity. These findings provide important new insights into the nature of recall responses to *C. burnetii*, warrant future studies to determine whether innate responses contribute to protection from *C. burnetii* infection and have the potential to inform the design of novel vaccines for Q fever.

## Data availability statement

The raw data supporting the conclusions of this article will be made available by the authors, without undue reservation.

## Ethics statement

The studies involving humans were approved by Medical Ethical Committee Brabant (Tilburg, Netherlands, NL51305.028.15). The studies were conducted in accordance with the local legislation and institutional requirements. The participants provided their written informed consent to participate in this study.

## Author contributions

SRP, AS, PR, AG, AES and MP conceptualized and designed the study and experiments. Experiments were performed by SRP and AS. Data were analyzed by SP, AS, SK, RS, JH, and RD, and interpreted by SRP and AS. AG, AES and MP acquired funding and supervised research activities. SRP and AS wrote the manuscript and PR, AES, AG and MP critically reviewed and approved the manuscript. All authors contributed to the article and approved the submitted version.
