# Supplementary material for: Cytometry profiling of ex vivo recall responses to Coxiella burnetii in previously naturally exposed individuals reveals long-term changes in both adaptive and innate immune cellular compartments
Source: Front Immunol. 2023 Oct 11;14:1249581. doi: 10.3389/fimmu.2023.1249581 (PMC10598782; doi:10.3389/fimmu.2023.1249581)
Supplement: Supplementary file 1 [file DataSheet_1.pdf]

1 1. Supplementary Figures and Tables

1.1 Supplementary Figures

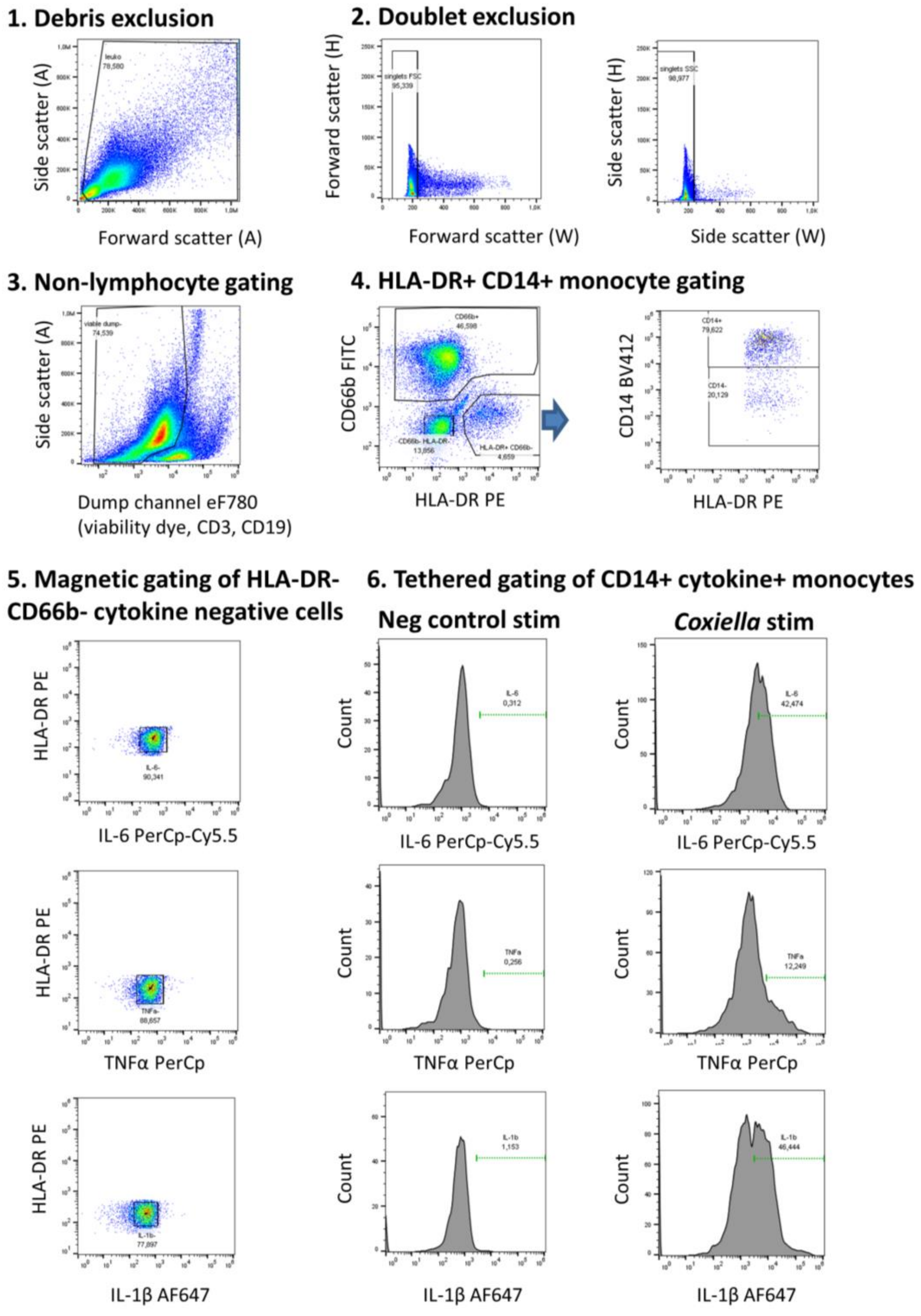

Supplementary Figure S1: Flow cytometry gating strategy used to identify monocytes and subsequently the proportion of cytokine-producing monocytes.

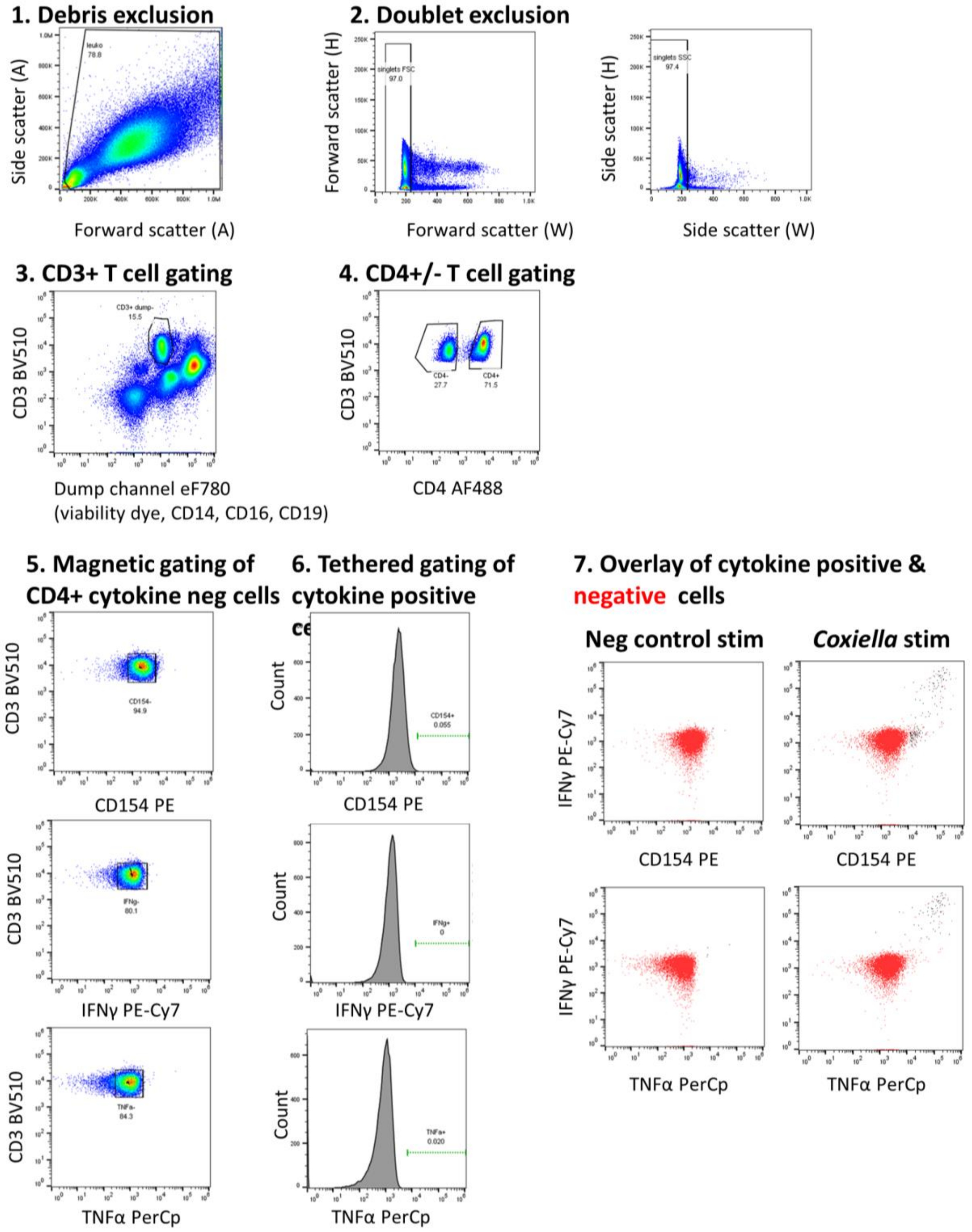

Supplementary Figure S2: Flow cytometry gating strategy used to identify T cells and subsequently the proportion of cytokine-producing T cells.

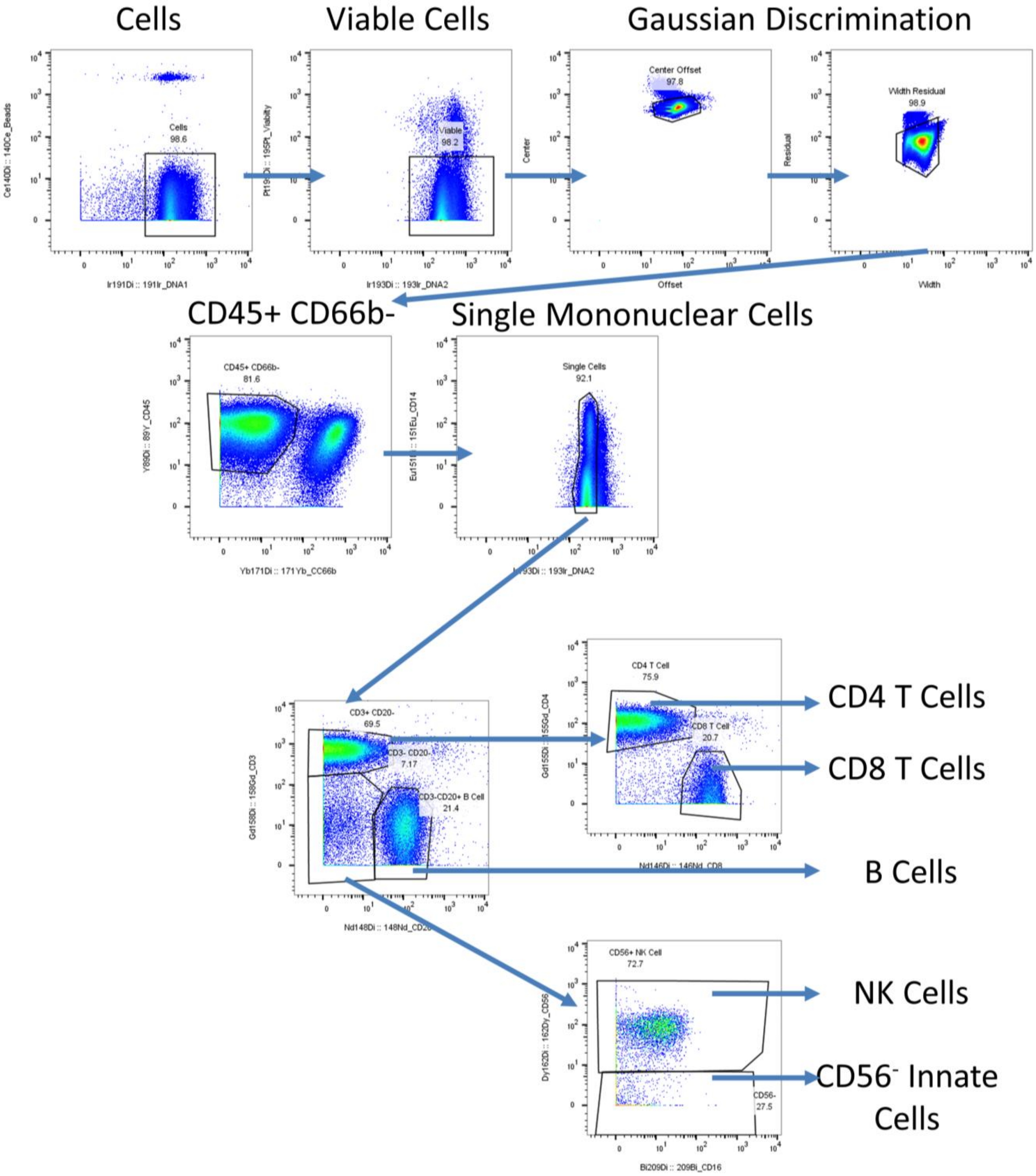

Supplementary Figure S3: Mass cytometry gating strategy used to identify major immune cell populations.

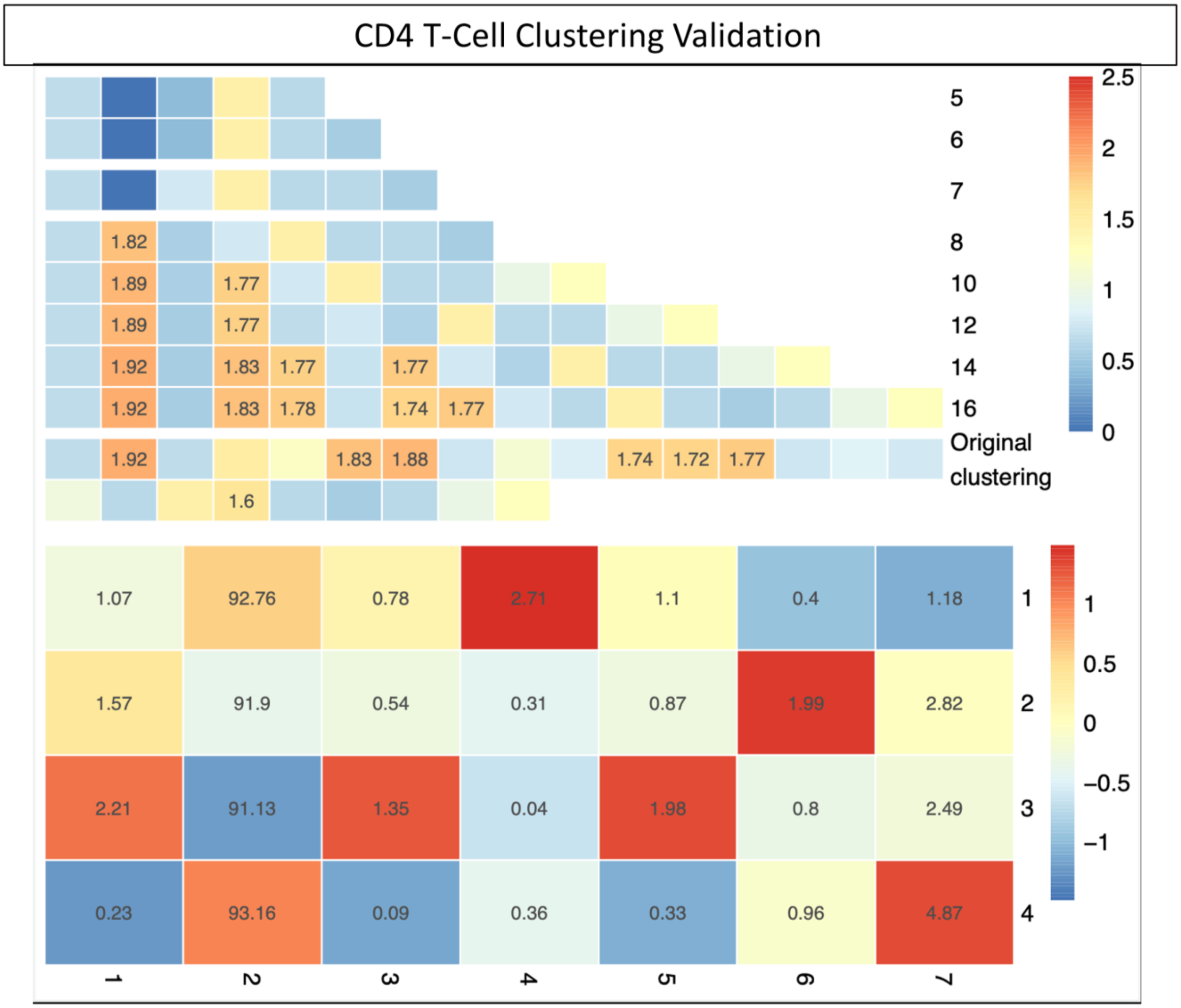

Supplementary Figure S4: Parameter optimization for FlowSOM clustering step of the CytoNorm algorithm for normalization of CD4$^+$ T cells. Seven metaclusters were chosen as the final number of metaclusters for CytoNorm based on coefficient of variation (CV) values within each cluster over a range of resulting metaclusters (top, CV values within each square of the grid). CV values over a value of 1.5 are shown, and the final number of metaclusters was chosen to be the highest number of metaclusters that a CV value did not exceed the 1.5 threshold. (Bottom) Shown are z-scored frequencies of each experimental group within the resulting CytoNorm metaclusters used for normalization.

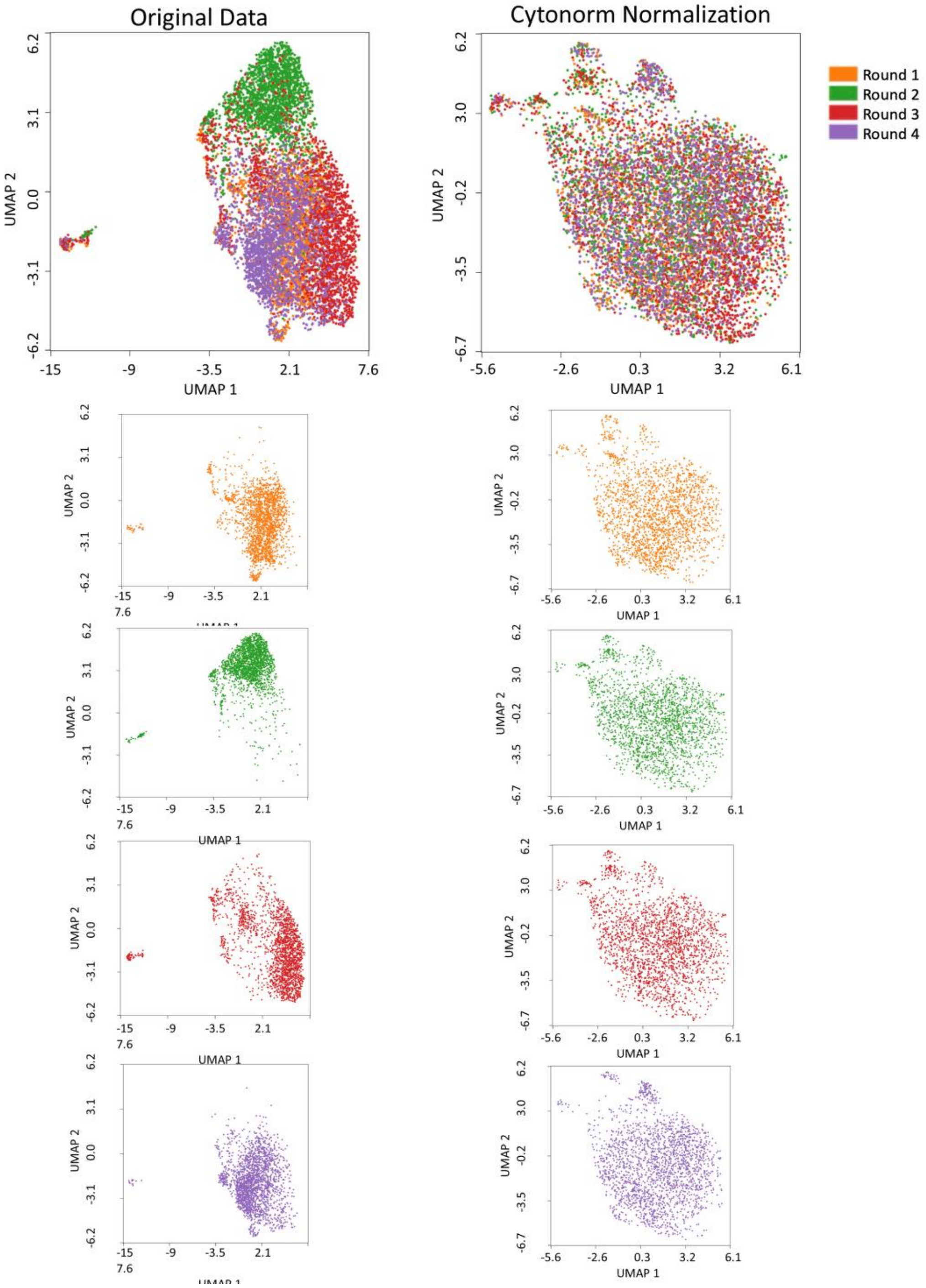

Supplementary Figure S5: Unstimulated reference CD4 T cell files used in rounds 1-4 of mass cytometry staining underwent dimension reduction using UMAP before and after CytoNorm normalization. Dimension reduction was completed using the 27 markers (see Methods) and UMAP settings: Fifteen neighbors and 0.4 minimum distance. The Original Data panel shows the unstimulated CD4 T cell files in rounds 1-4 prior to CytoNorm normalization, with the top plot showing all four files overlaid, and the four lower plots displaying each individual file. The CytoNorm Normalized panel shows the same four unstimulated CD4 T cell reference files following CytoNorm normalization, with the top plot displaying all four files overlaid, and the four lower plots displaying each individual file.

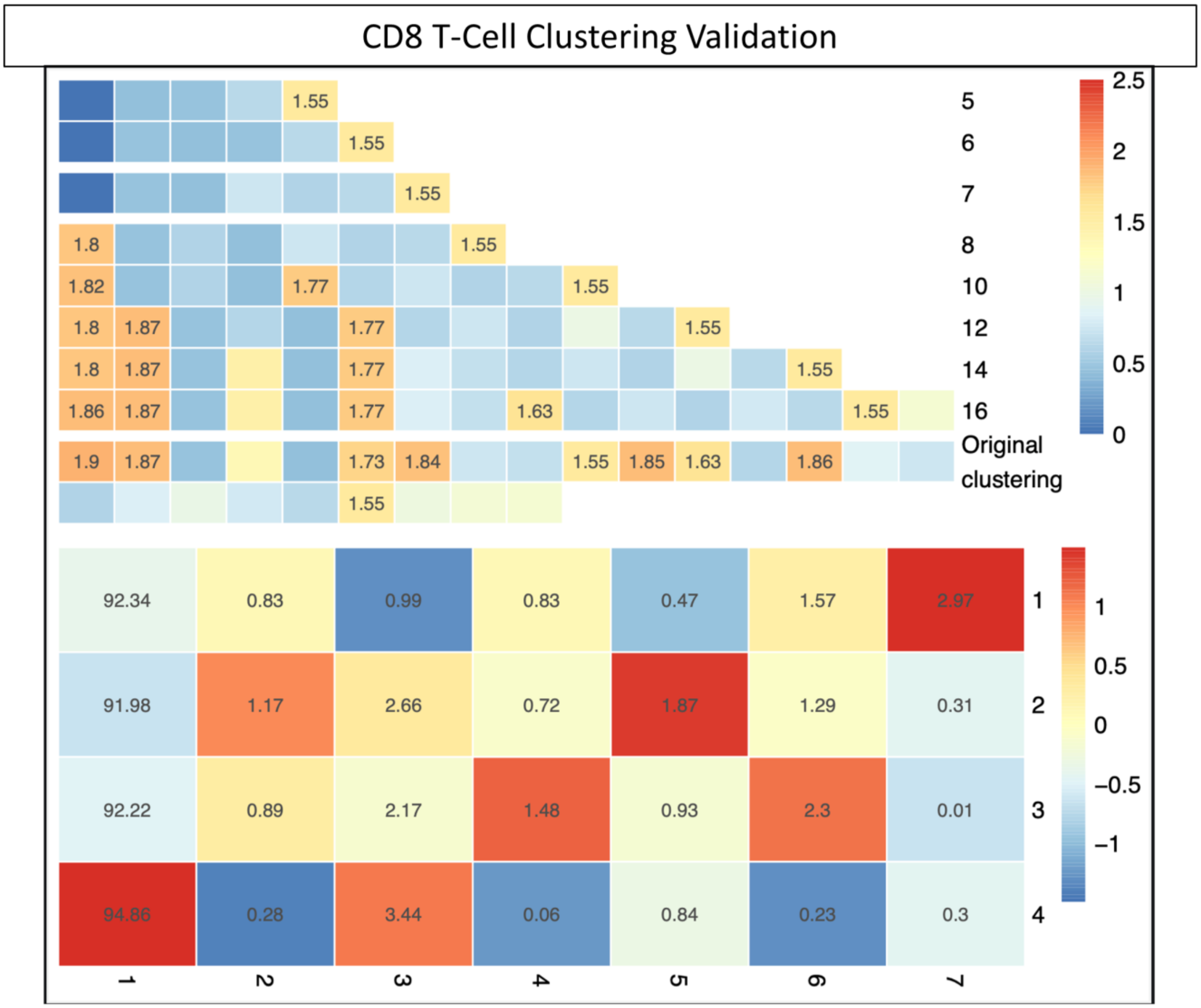

Supplementary Figure S6: Parameter optimization for FlowSOM clustering step of CytoNorm algorithm for normalization of CD8$^+$ T cells. In contrast to the normalization of all other cell populations, even low numbers of metaclusters contained a single cluster with a coefficient of variation (CV) greater than 1.5. Seven metaclusters were chosen as the final number of clusters for CytoNorm based on coefficient of variation (CV) values within each cluster over a range of resulting metaclusters (top, CV values within each square of the grid). CV values over a value of 1.5 are shown, and the final number of metaclusters was chosen to be 7, the highest number of metaclusters that a CV value did not exceed the 1.5 threshold (excluding the one cluster that was present at all numbers of clusters). (Bottom) Shown are z-scored frequencies of each experimental group within the resulting CytoNorm metaclusters used for normalization.

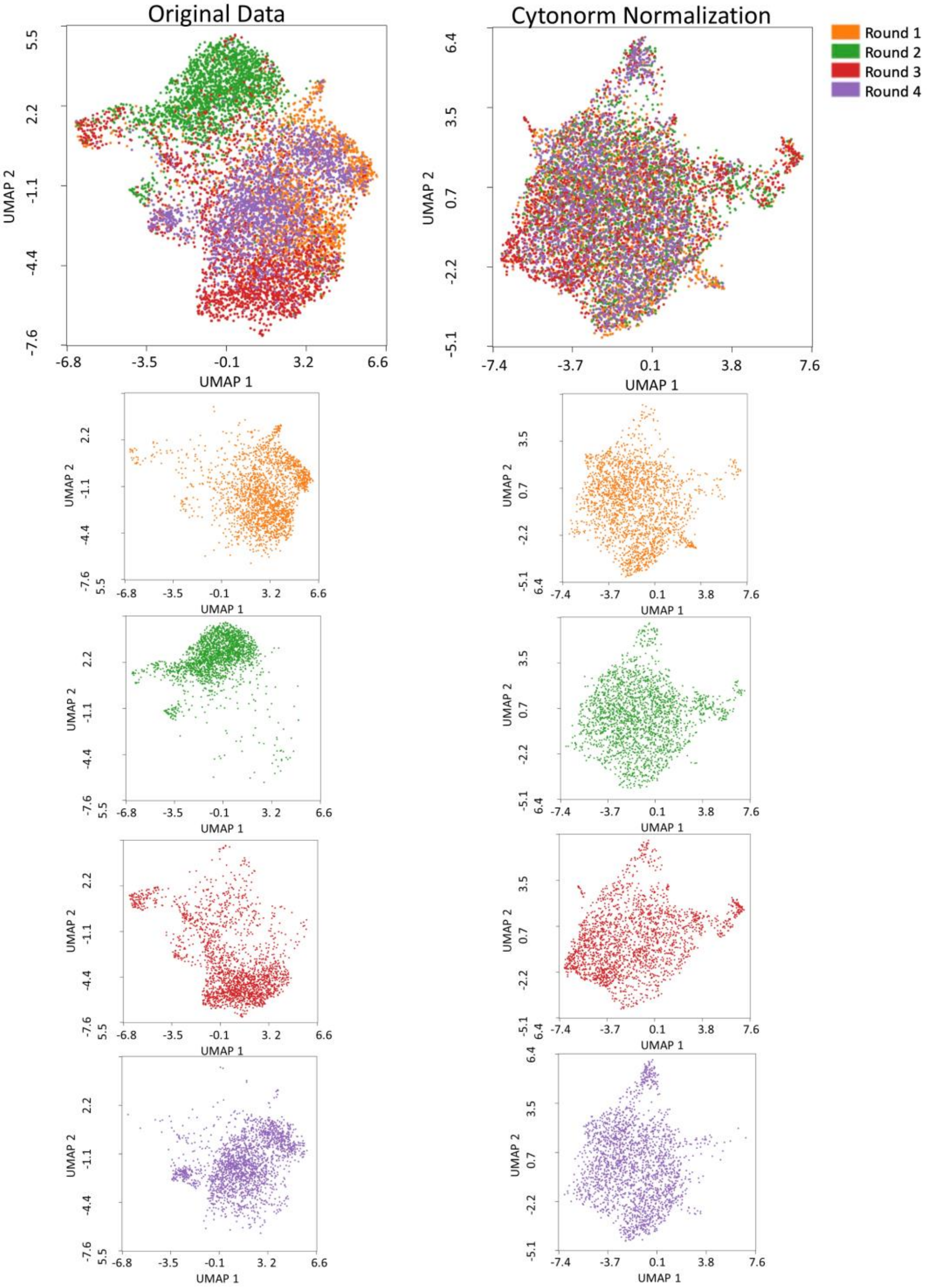

Supplementary Figure S7: Unstimulated reference CD8 T cell files used in rounds 1-4 of mass cytometry staining underwent dimension reduction using UMAP before and after CytoNorm normalization. Dimension reduction was completed using the 27 markers (see Methods) and UMAP settings: Fifteen neighbors and 0.4 minimum distance. The Original Data panel shows the unstimulated CD8 T cell files in rounds 1-4 prior to CytoNorm normalization, with the top plot showing all four files overlaid, and the four lower plots displaying each individual file. The CytoNorm Normalized panel shows the same four unstimulated CD8 T cell reference files following CytoNorm normalization, with the top plot displaying all four files overlaid, and the four lower plots displaying each individual file.

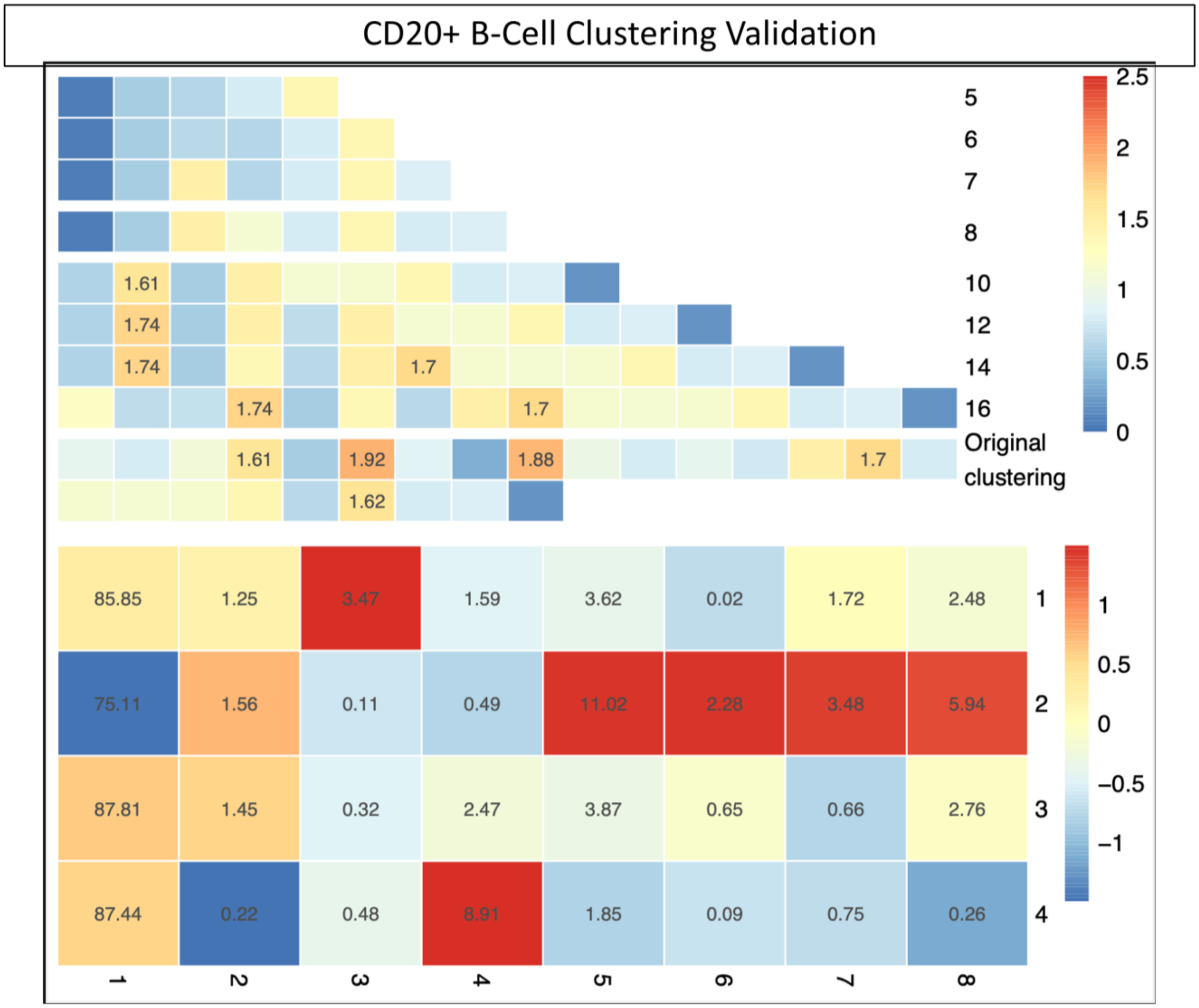

Supplementary Figure S8: Parameter optimization for FlowSOM clustering step of CytoNorm algorithm for normalization of $CD20^+$ B cells. Eight metaclusters were chosen as the final number of clusters for CytoNorm based on coefficient of variation (CV) values within each cluster over a range of resulting metaclusters (top, CV values within each square of the grid). CV values over a value of 1.5 are shown, and the final number of metaclusters was chosen to be 8, the highest number of clusters that the CV of each metacluster did not exceed the 1.5 threshold. (Bottom) Shown are z-scored frequencies of each experimental group within the resulting CytoNorm metaclusters used for normalization.

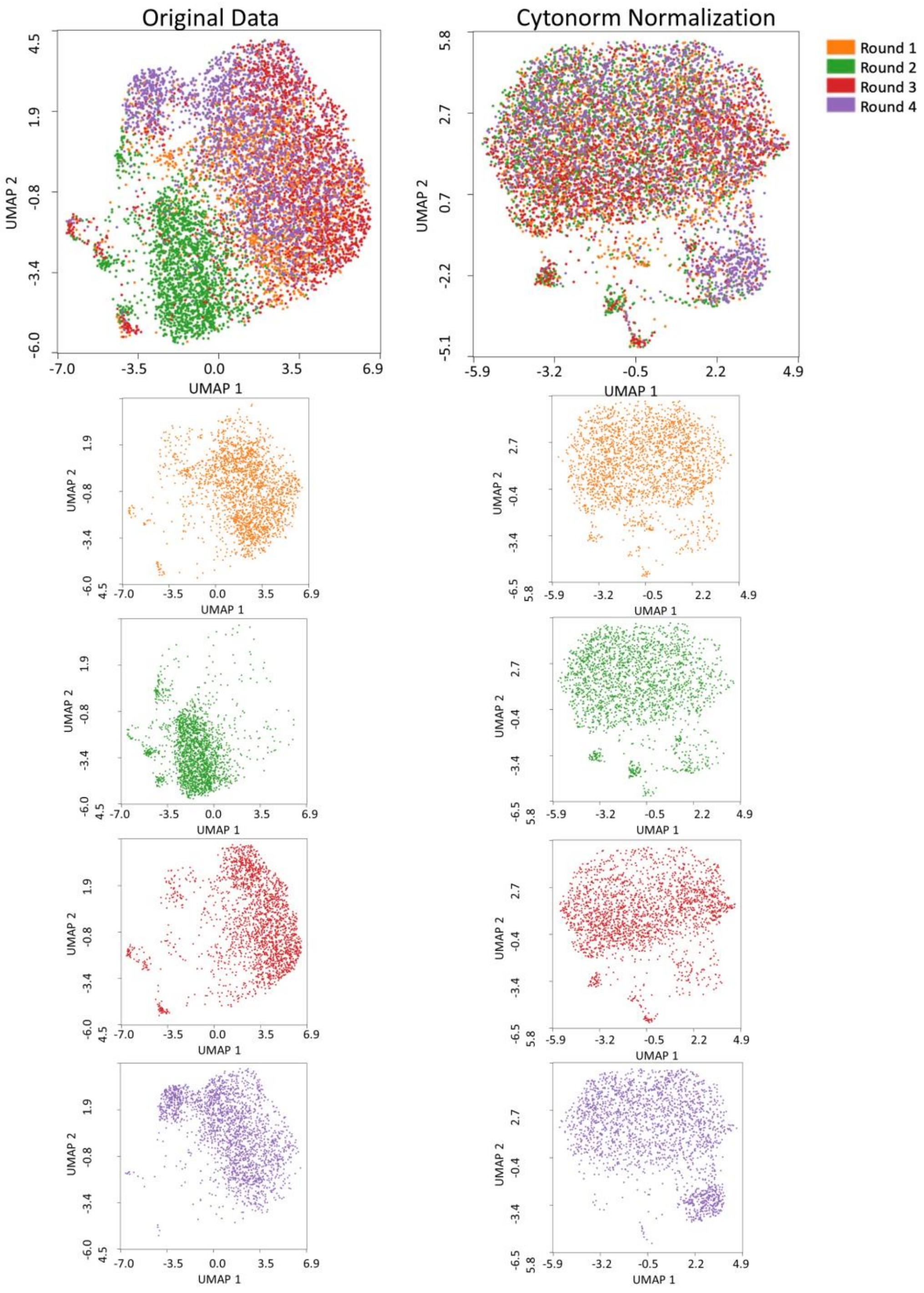

Supplementary Figure S9: Unstimulated reference CD20$^+$ B cell files used in rounds 1-4 of mass cytometry staining underwent dimension reduction using UMAP before and after CytoNorm normalization. Dimension reduction was completed using the 27 markers (see Methods) and UMAP settings: Fifteen neighbors and 0.4 minimum distance. The Original Data panel shows the unstimulated CD20$^+$ B cell files in rounds 1-4 prior to CytoNorm normalization, with the top plot showing all four files overlaid, and the four lower plots displaying each individual file. The CytoNorm Normalized panel shows the same four unstimulated CD20$^+$ B cell reference files following CytoNorm normalization, with the top plot displaying all four files overlaid, and the four lower plots displaying each individual file.

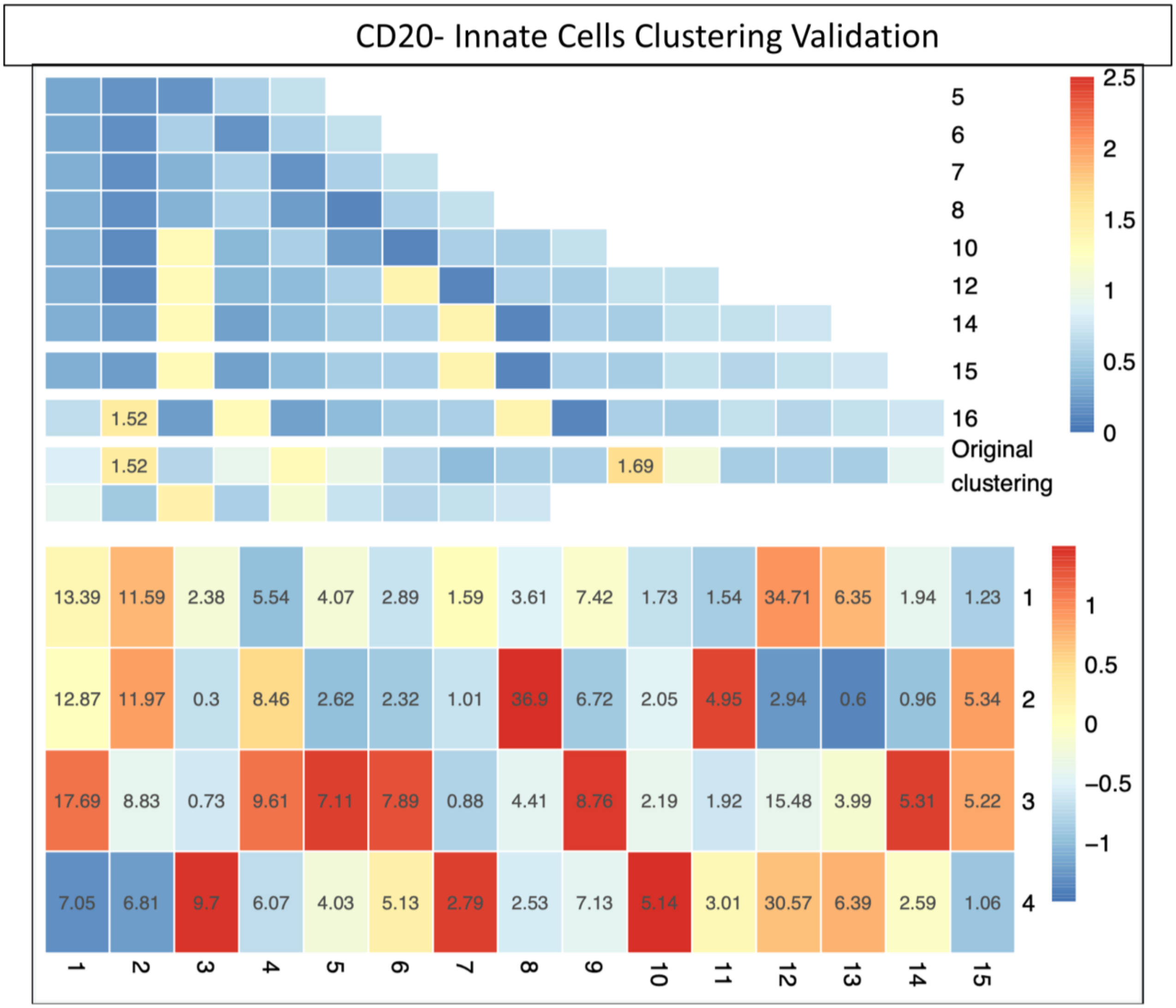

Supplementary Figure S10: Parameter optimization for FlowSOM clustering step of CytoNorm algorithm for normalization of CD20$^-$ innate cells. In contrast to the normalization of all other cell populations, low numbers of resulting metaclusters contained a single cluster with a coefficient of variation (CV) greater than 1.5. Fifteen metaclusters were chosen as the final number of clusters for CytoNorm based on coefficient of variation (CV) values within each cluster over a range of resulting metaclusters (top, CV values within each square of the grid). CV values over a value of 1.5 are shown, and the final number of metaclusters was chosen to be 15, the highest number of clusters that the CV of each metacluster did not exceed the 1.5 threshold. (Bottom) Shown are z-scored frequencies of each experimental group within the resulting CytoNorm metaclusters used for normalization

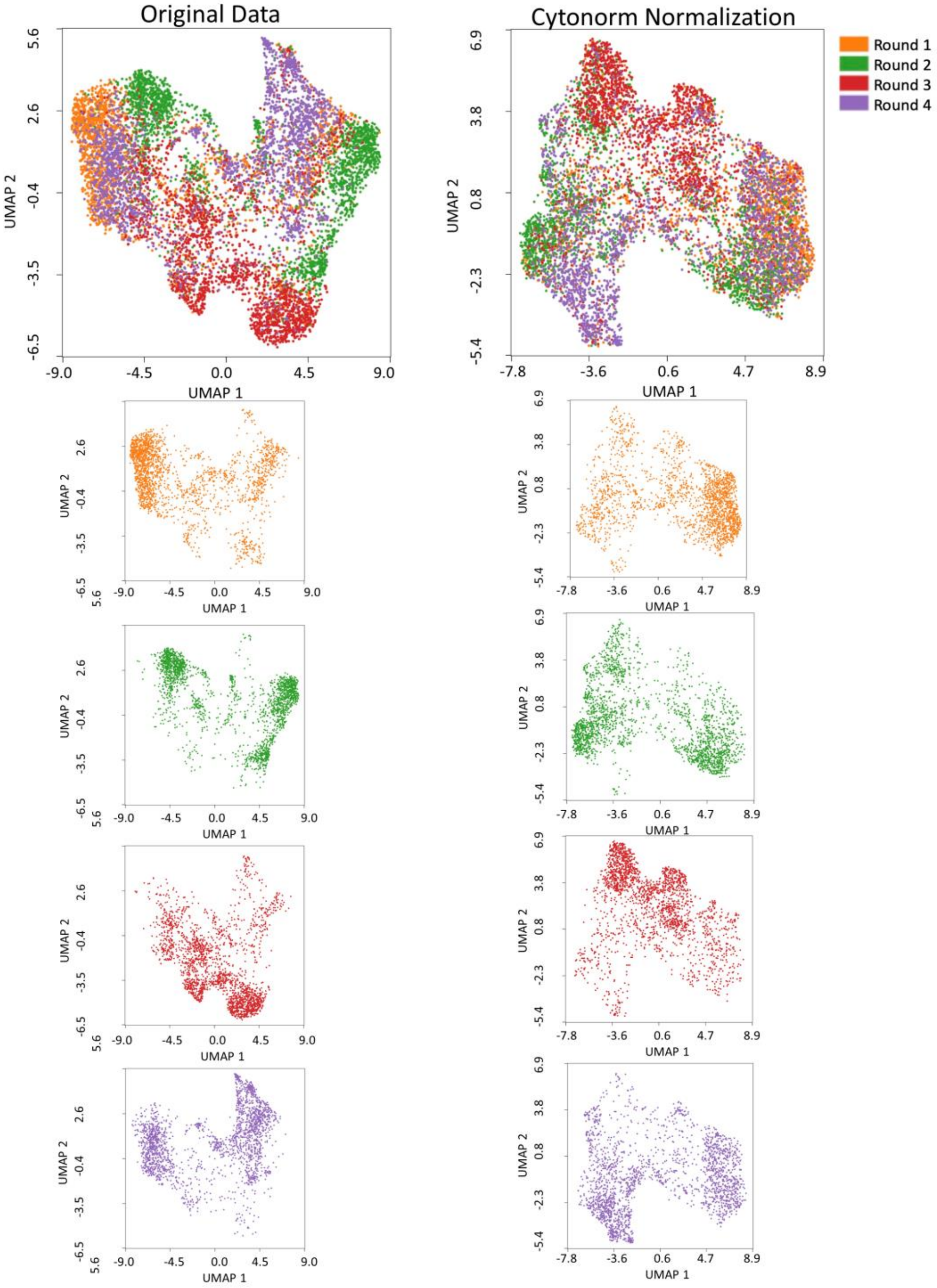

Supplementary Figure S11: Unstimulated reference CD20^{-} innate Cell files used in rounds 1-4 of mass cytometry staining underwent dimension reduction using UMAP before and after CytoNorm normalization. Dimension reduction was completed using the 27 markers (see Methods) and UMAP settings: Fifteen neighbors and 0.4 minimum distance. The Original Data panel shows the unstimulated CD20^{-} innate cell files in rounds 1-4 prior to CytoNorm normalization, with the top plot showing all four files overlaid, and the four lower plots displaying each individual file. The CytoNorm Normalized panel shows the same four unstimulated CD20^{-} innate cell reference files following CytoNorm normalization, with the top plot displaying all 4 files overlaid, and the four lower plots displaying each individual file.

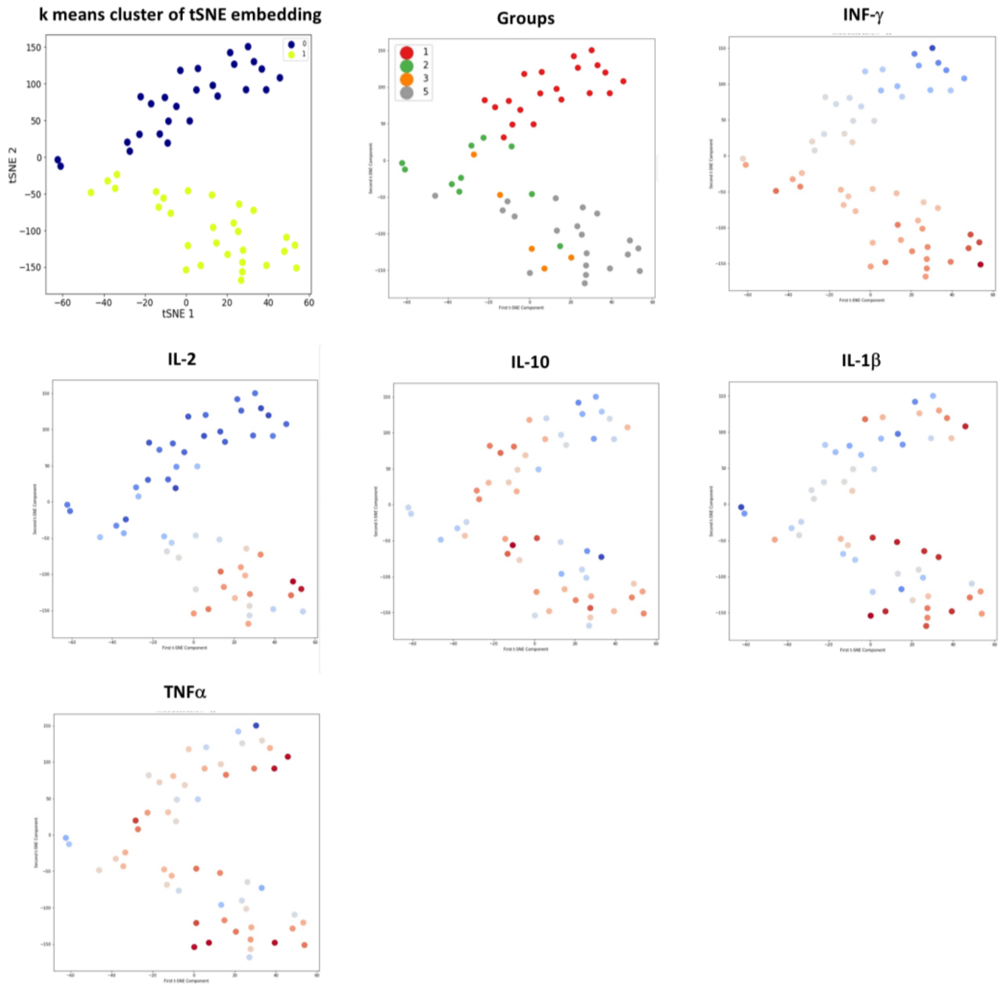

Supplementary Figure S12: Identification of cytokine clusters by high dimensional reduction and k-means clustering of five cytokine variables. Scatter plot showing tSNE projections: The x and y axes represents the two tSNE coordinates after tSNE dimension reduction of all donors based on their cytokine concentrations. Each point in the plot represents an individual sample. (Top row, left) The color depicts which cluster the sample belongs to and in (top row, middle) color depicts which group the individual was assigned to. The remaining scatter plots: Each point in the plot represents an individual sample. The color gradient indicates from blue to red, low to high, the background corrected concentration of each cytokine. The data are min-max transformed for visualization.

1.2 Supplementary Tables

Supplementary Table 1: Correlation Coefficients (Spearman Rho) indicating correlation between cytokine levels in plasma and background corrected cluster abundances.

	IGRA 2015	IFNγ	IL-10	IL-1β	IL-2	TNFα
CD4 Cluster 1	-0.06	-0.08	-0.01	0.09	0.14	0.41
CD4 Cluster 10	-0.19	-0.19	0.00	0.14	-0.24	0.14
CD4 Cluster 11	0.68	0.55	0.28	0.18	0.68	0.11
CD4 Cluster 12	0.58	0.45	0.30	0.33	0.65	0.22
CD4 Cluster 13	0.03	-0.05	-0.13	0.05	-0.09	-0.06
CD4 Cluster 14	-0.05	0.07	-0.08	-0.10	0.07	-0.03
CD4 Cluster 15	0.06	0.15	0.06	-0.28	0.14	0.01
CD4 Cluster 16	0.15	0.13	0.17	0.05	0.24	0.16
CD4 Cluster 17	-0.34	-0.34	0.07	-0.09	-0.16	0.12
CD4 Cluster 18	-0.34	-0.21	0.18	-0.02	-0.24	-0.17
CD4 Cluster 19	0.47	0.36	-0.10	0.22	0.49	0.21
CD4 Cluster 2	-0.35	-0.32	0.08	0.02	-0.13	0.37
CD4 Cluster 20	-0.27	-0.21	0.14	-0.06	-0.08	0.18
CD4 Cluster 3	-0.32	-0.33	-0.22	-0.13	-0.32	0.12
CD4 Cluster 4	0.42	0.27	-0.36	-0.13	0.33	-0.04
CD4 Cluster 5	-0.23	-0.14	-0.10	-0.14	-0.36	-0.47
CD4 Cluster 6	0.48	0.37	0.19	0.10	0.52	0.19

CD4 Cluster 7	-0.01	0.00	-0.25	0.11	-0.03	0.02
CD4 Cluster 8	0.36	0.25	-0.08	0.12	0.13	-0.11
CD4 Cluster 9	0.09	-0.14	-0.18	-0.28	0.11	-0.06
CD8 Cluster 1	-0.11	-0.07	-0.02	-0.02	0.07	0.38
CD8 Cluster 10	-0.20	-0.24	-0.13	-0.26	-0.21	0.07
CD8 Cluster 11	0.64	0.50	0.23	0.27	0.48	0.02
CD8 Cluster 12	0.09	0.11	-0.09	0.06	0.04	-0.15
CD8 Cluster 13	0.22	0.28	0.21	-0.06	0.24	-0.01
CD8 Cluster 14	0.08	-0.03	0.22	0.09	0.15	0.16
CD8 Cluster 15	0.20	0.20	0.17	-0.05	0.22	-0.11
CD8 Cluster 16	0.15	0.09	-0.08	-0.09	-0.13	-0.21
CD8 Cluster 17	-0.09	-0.15	0.09	-0.05	-0.08	0.15
CD8 Cluster 18	0.30	0.38	-0.29	-0.02	0.14	-0.21
CD8 Cluster 19	-0.05	-0.15	-0.28	-0.10	0.01	0.01
CD8 Cluster 2	-0.35	-0.23	-0.04	-0.10	-0.14	0.22
CD8 Cluster 20	0.29	0.28	-0.12	0.02	0.34	0.09
CD8 Cluster 3	-0.38	-0.26	0.00	-0.08	-0.17	0.28
CD8 Cluster 4	0.30	0.25	-0.21	-0.14	0.32	-0.01
CD8 Cluster 5	0.55	0.43	0.12	0.17	0.37	-0.01
CD8 Cluster 6	-0.25	-0.19	-0.01	-0.14	-0.31	-0.46

CD8 Cluster 7	0.30	0.21	-0.02	0.11	0.34	0.12
CD8 Cluster 8	0.34	0.28	-0.37	0.00	0.19	-0.22
CD8 Cluster 9	0.06	-0.01	0.25	0.05	0.14	0.35
B Cell Cluster 1	-0.11	-0.05	-0.37	-0.24	-0.29	-0.31
B Cell Cluster 10	0.24	0.28	0.04	0.21	0.12	0.25
B Cell Cluster 11	0.21	0.02	0.14	-0.25	0.02	-0.22
B Cell Cluster 12	-0.16	-0.14	-0.09	0.09	0.00	0.23
B Cell Cluster 13	0.32	0.39	0.08	0.47	0.43	0.22
B Cell Cluster 14	0.15	0.13	0.02	-0.09	0.12	-0.33
B Cell Cluster 15	0.02	0.02	0.24	0.36	0.25	0.51
B Cell Cluster 16	0.16	0.19	0.07	0.34	0.38	0.44
B Cell Cluster 17	-0.26	-0.31	0.12	-0.26	-0.39	-0.14
B Cell Cluster 18	-0.09	-0.07	0.11	-0.23	-0.18	-0.08
B Cell Cluster 19	-0.21	-0.20	0.17	0.17	-0.02	0.51
B Cell Cluster 2	0.34	0.31	-0.21	0.27	0.28	0.13
B Cell Cluster 20	0.29	0.14	-0.01	0.21	0.37	0.48
B Cell Cluster 3	0.20	0.07	0.34	0.36	0.46	0.37
B Cell Cluster 4	-0.08	0.00	-0.12	-0.33	-0.15	-0.33
B Cell Cluster 5	0.30	0.31	0.31	0.02	0.19	-0.22
B Cell Cluster 6	-0.15	-0.15	-0.18	0.08	-0.05	0.19

B Cell Cluster 7	-0.11	-0.21	0.08	-0.40	-0.16	-0.27
B Cell Cluster 8	0.15	0.03	0.13	-0.24	0.00	-0.29
B Cell Cluster 9	0.01	0.02	0.24	0.36	0.26	0.50
Innate Cell Cluster 1	0.16	0.09	-0.48	-0.14	0.00	-0.20
Innate Cell Cluster 10	-0.27	-0.25	-0.43	-0.23	-0.17	-0.10
Innate Cell Cluster 11	0.27	0.25	0.45	0.28	0.28	0.42
Innate Cell Cluster 12	-0.31	-0.42	-0.24	-0.20	-0.26	0.12
Innate Cell Cluster 13	-0.28	-0.23	-0.26	-0.09	-0.36	-0.05
Innate Cell Cluster 14	-0.52	-0.48	0.09	-0.02	-0.39	0.36
Innate Cell Cluster 15	0.04	0.04	-0.05	-0.04	-0.12	-0.18
Innate Cell Cluster 16	-0.47	-0.28	0.14	-0.05	-0.27	0.00
Innate Cell Cluster 17	0.38	0.39	0.07	0.00	0.29	-0.08
Innate Cell Cluster 18	-0.53	-0.38	0.00	-0.06	-0.33	0.10
Innate Cell Cluster 19	0.62	0.66	0.02	0.23	0.61	0.28
Innate Cell Cluster 2	-0.01	-0.03	0.61	0.28	0.11	0.19
Innate Cell Cluster 20	-0.13	-0.01	0.14	-0.10	-0.11	0.07
Innate Cell Cluster 3	-0.52	-0.50	0.36	-0.05	-0.26	0.29
Innate Cell Cluster 4	0.09	0.12	0.70	0.29	0.20	0.30
Innate Cell Cluster 5	0.55	0.29	0.16	0.17	0.40	0.19
Innate Cell Cluster 6	-0.30	-0.22	-0.09	-0.10	-0.28	0.04

Innate Cell Cluster 7	0.63	0.61	0.08	0.10	0.48	-0.17
Innate Cell Cluster 8	-0.13	0.12	-0.05	-0.08	-0.10	-0.26
Innate Cell Cluster 9	0.22	0.24	-0.30	-0.04	0.21	-0.20
